# Supplementary material for: Liver-secreted fluorescent blood plasma markers enable chronic imaging of the microcirculation
Source: Cell Rep Methods. 2022 Sep 21;2(10):100302. doi: 10.1016/j.crmeth.2022.100302 (PMC9606131; doi:10.1016/j.crmeth.2022.100302)
Supplement: Document S1. Figures S1–S5 [file mmc1.pdf]

Cell Reports Methods, Volume *2*

Supplemental information

Liver-secreted fluorescent blood plasma markers enable chronic imaging of the microcirculation

Xiaowen Wang, Christine Delle, Antonis Asiminas, Sonam Akther, Marta Vittani, Peter Brøgger, Peter Kusk, Camilla Trang Vo, Tessa Radovanovic, Ayumu Konno, Hirokazu Hirai, Masahiro Fukuda, Pia Weikop, Steven A. Goldman, Maiken Nedergaard, and Hajime Hirase

Supplemental information

Liver-secreted fluorescent blood plasma markers enable chronic imaging of the microcirculation

Xiaowen Wang[1+], Christine Delle[1+], Antonis Asiminas[1+], Sonam Akther[1], Marta Vittani[1], Peter Brøgger[1], Peter Kusk[1], Camilla Trang Vo[1], Tessa Radovanovic[1], Ayumu Konno[2,3], Hirokazu Hirai[2,3], Masahiro Fukuda[4,5], Pia Weikop[1], Steven A Goldman[1,6], Maiken Nedergaard[1,6*], Hajime Hirase[1,6,7*]

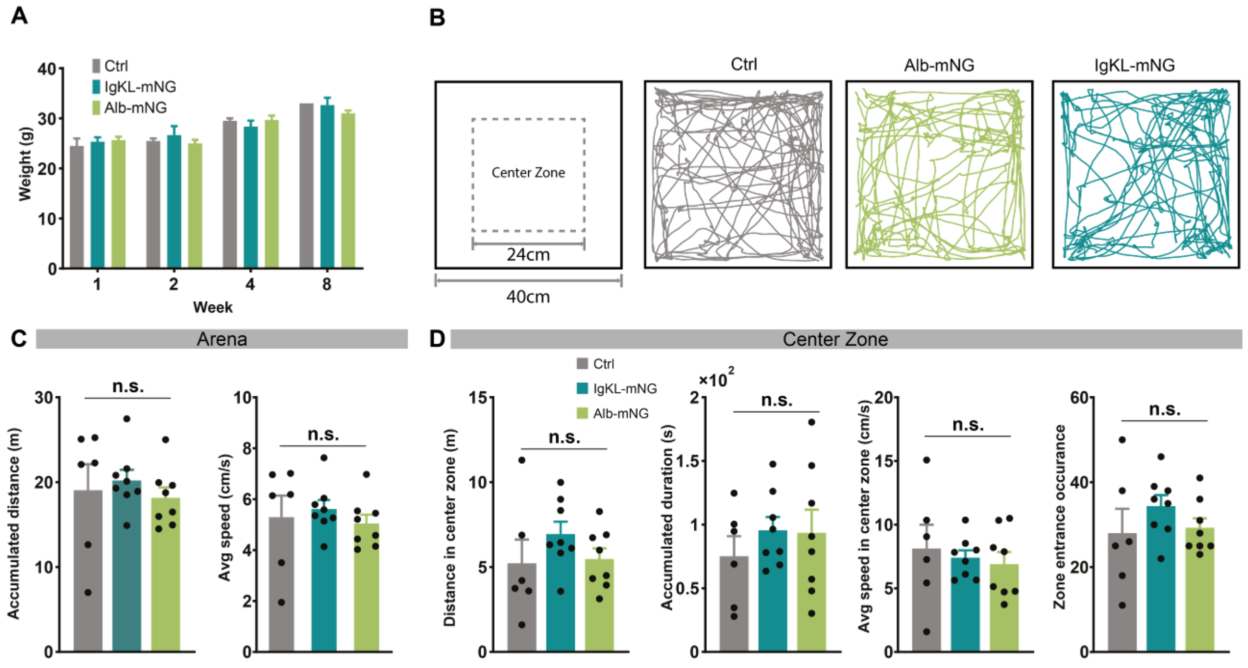

Figure S1. Plasma tracer expression does not display obvious phenotypes in body weight or open field behavior. Related to Figure 2. (A) Body weight of control (age matched sham) and AAV-P3-IgKL-mNG-injected and AAV8-P3-Alb-mNG-injected mice during 1 to 8 weeks post-injection. AAV-injected mice show no differences in body weight compared to control; two-way ANOVA: significant main effect of time, no significant effect of group, or group x time interaction; n=3 mice per group. (B) Schematic of the arena used for open field test and example traces of mouse trajectory for the last 6 min of 10 min recording. (C) Total distance traveled (left) and mean speed of movement (right) during the last 6 min of open field behavior; one-way ANOVA: no significant main effect of group; n=6–8 mice per group. (D) Metrics on center zone behavior. Distance moved, total time, speed of movement, and frequency of visiting the center zone did not show significant differences among control and AAV injected mice; one-way ANOVA: no significant main effect of group for all metrics $p>0.05$; n=6–8 mice per group. All graphs show means ± SEM.

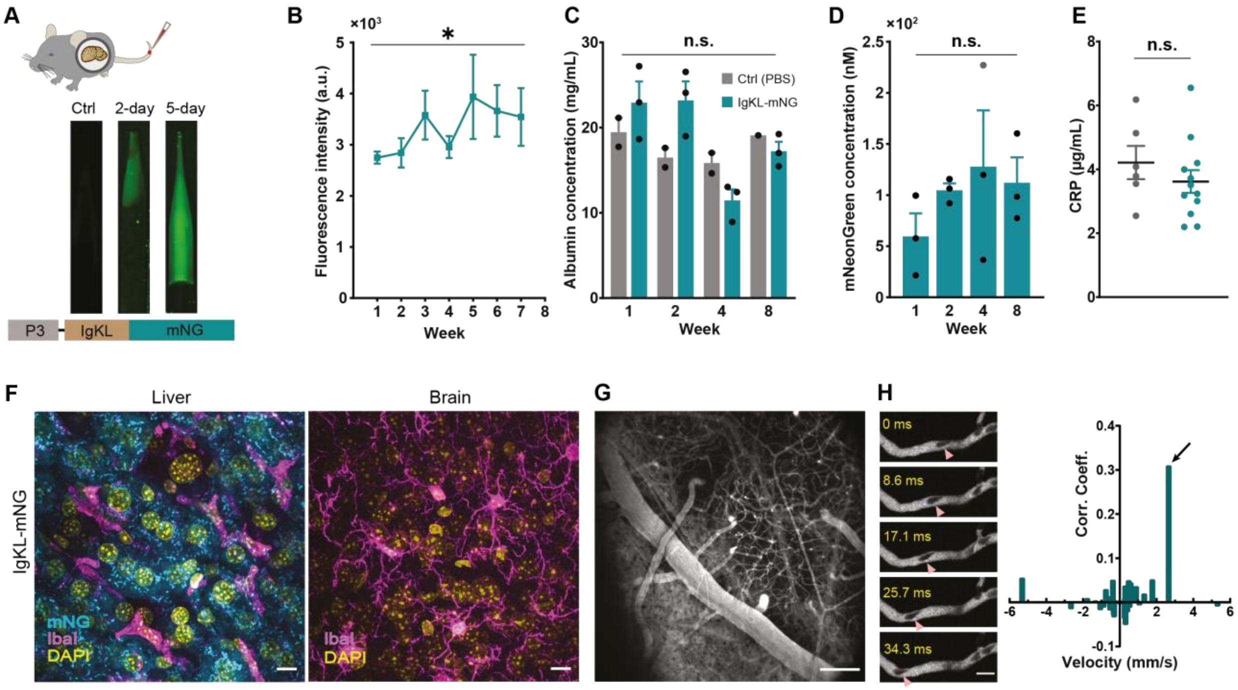

Figure S2. Liver-targeted expression IgKL-mNG (secretory mNG). Related to Figure 2. (A) A secretory form of mNeonGreen, IgKL-mNG, is expressed in the liver by systemic injection of AAV8-P3-IgKL-mNG in mice. Fluorescence signals were detected in the blood samples two days after AAV injection. (B) Chronic monitoring of plasma fluorescence. Note that the plasma intensity is an order of magnitude lower than Alb-mNG (Fig. 2) (one-way ANOVA: significant effect of time, $p < 0.05$); n=6 mice. (C) Plasma albumin concentration and plasma mNG concentration (D) over eight weeks; Albumin concentration: two-way ANOVA: no significant effect of time, group or interaction; mNG concentration: one-way ANOVA: no significant effect of time; n=3 mice. (E) CRP levels during the 8 weeks of post-AAV injection period is indistinguishable from sham-injected control. (t-test, $p > 0.05$; $n_{control}=6$, $n_{IgKL\text{-}mNG}=12$. (F) Liver and brain images 3 weeks after AAV injection. Immunofluorescence: mNG (cyan), IBA1 (mangenta), DAPI (yellow). Scale bar 10 µm. (G) Two-photon imaging through a cranial window visualizes cerebral blood vasculature despite the relatively low fluorescence signal intensity. (H) Capillary blood flow is also quantifiable using IgKL-mNG as a plasma tracer (RBC speed = 2.65 mm/s). All graphs show means ± SEM; * $p < 0.05$.

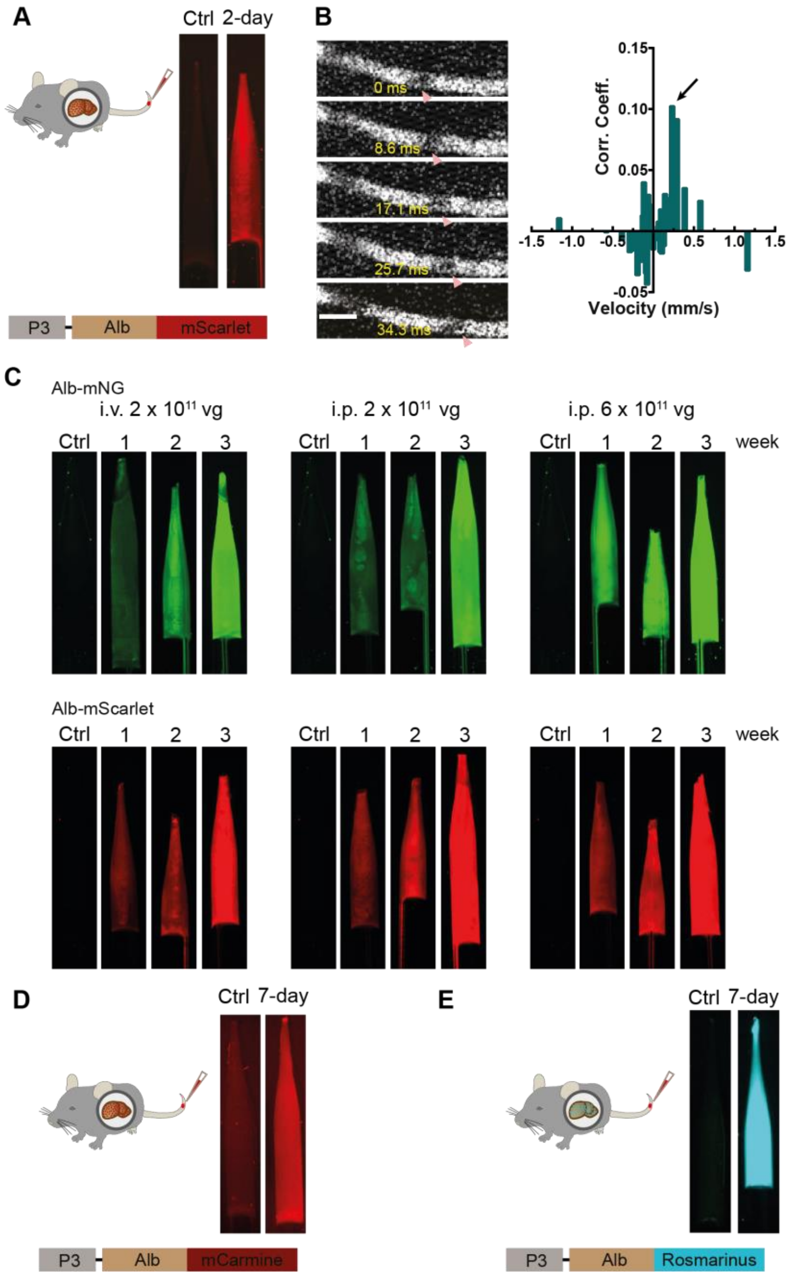

Figure S3. Expanding the spectrum of liver-secreted plasma fluorescent probes. Related to Figures 2, 3 and 4. (A) Retro-orbital i.v. injection of AAV8-P3-Alb-mScarlet results in labeling of blood plasma with red fluorescence, thereby representing a plasma tracer that is spectrally distinct from Alb-mNG. (B) Capillary flow dynamics is reliably visualized by two-photon microscopy (RBC speed = 0.25 mm/s). (C) Samples of fluorescent blood plasma taken from animals that were injected either i.v. or i.p. (at two different concentrations) with either AAV8-P3-Alb-mNG or AAV8-P3-Alb-mScarlet 1, 2, and 3 weeks after injection. (D–E) Alb-based plasma tracer spectrum is further extended by the addition of and Alb-mCarmine (D: deep red fluorescence), and Alb-Rosmarinus (E: cyan fluorescence). For both, example of the fluorescence signals in blood samples collected on day 7 after a single retro-orbital i.v. injection of either AAV8-P3-Alb-mCarmine or AAV8-P3-Alb-Rosmarinus.

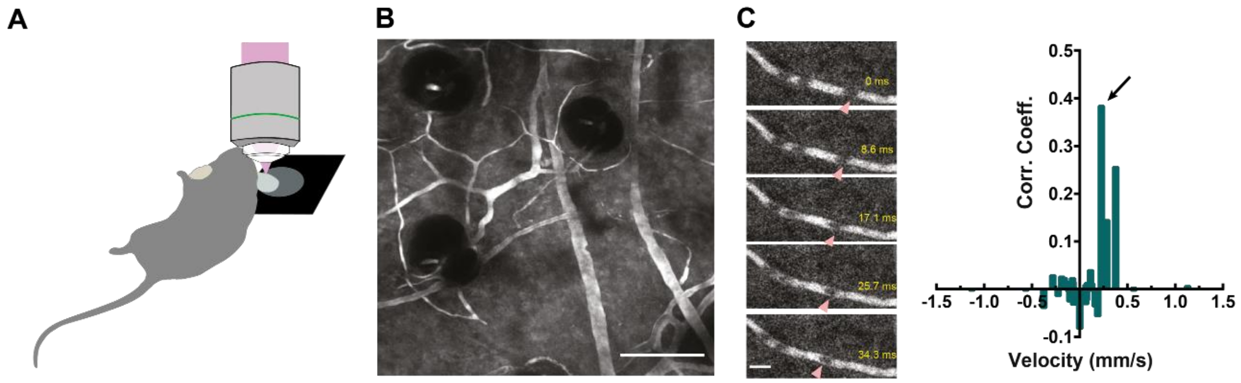

Figure S4. Alb-mNG is suitable for studying vasculature in peripheral tissues. Related to Figure 3. (A) Schematic of two-photon imaging of the ear skin capillary network in an Alb-mNG expressing mouse under ketamine-xylazine anesthesia. (B) Example image of ear vasculature. The black holes are the cavity space for hair follicle. Scale bar 100 µm (C) Measurement of blood flow in peripheral ear capillary via two-photon imaging (RBC speed = 0.23 mm/s). Scale bar 10 µm.

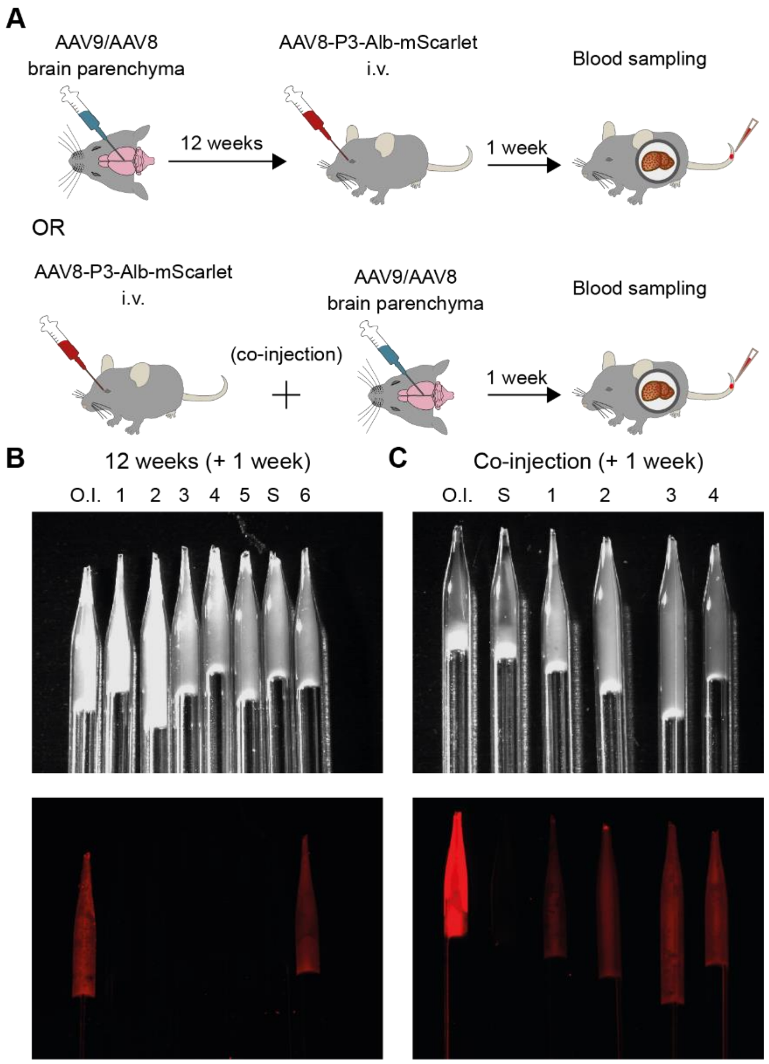

Figure S5. Expression of albumin-fused fluorescent probes is contigent on time after first AAV injection directly into brain parenchyma. Related to Figure 2 and 3. (A) Schematic of procedures. Mice were injected directly into parenchyma with AAV9-hSyn-GRAB$_{\mathrm{NE2m}}$ and AAV8-hSyn-GRAB$_{\mathrm{ACh3.0}}$ followed by retro-orbital administration (i.v.) of AAV8-P3-Alb-mScarlet 12 weeks after (top) or injected retro-orbitally (i.v.) with AAV8-P3-Alb-mScarlet and within 1 h were injected directly into parenchyma with AAV9-hSyn-GRAB$_{\mathrm{NE2m}}$ and AAV8-hSyn-GRAB$_{\mathrm{ACh3.0}}$ (bottom). (B) Bright-field (top) and red fluorescence images (bottom) of glass capillaries containing blood samples collected 1 week after i.v. injection of AAV8-P3-Alb-mScarlet proceeded by 12 weeks of brain parenchymal injection of AAV9-hSyn-GRAB$_{\mathrm{NE2m}}$ and AAV8-hSyn-GRAB$_{\mathrm{ACh3.0}}$ in five mice (1–5). A mouse injected with AAV8-P3-Alb-mScarlet 4 weeks before blood sampling (O.I.) and a mouse injected only with AAV8-P3-Alb-mScarlet (6) were used as positive controls. Blood from a saline-injected mouse was used as a negative control (S). (C) Bright-field (top) and red fluorescence images (bottom) of glass capillaries containing blood samples collected 1 week after co-injection of AAV8-P3-Alb-mScarlet (i.v.) and brain parenchymal AAV9-hSyn-GRAB$_{\mathrm{NE2m}}$ and AAV8-hSyn-GRAB$_{\mathrm{ACh3.0}}$ in four mice (1–4). A mouse injected with AAV8-P3-Alb-mScarlet 4 weeks before blood sampling (O.I.) was used as a positive control and blood from a saline-injected mouse was used as a negative control (S).
