# Supplementary material for: Liver-secreted fluorescent blood plasma markers enable chronic imaging of the microcirculation
Source: Cell Rep Methods. 2022 Sep 21;2(10):100302. doi: 10.1016/j.crmeth.2022.100302 (PMC9606131; doi:10.1016/j.crmeth.2022.100302)
Supplement: Document S2. Article plus supplemental information [file mmc2.pdf]

Cell Reports Methods

Report

Liver-secreted fluorescent blood plasma markers enable chronic imaging of the microcirculation

Graphical abstract

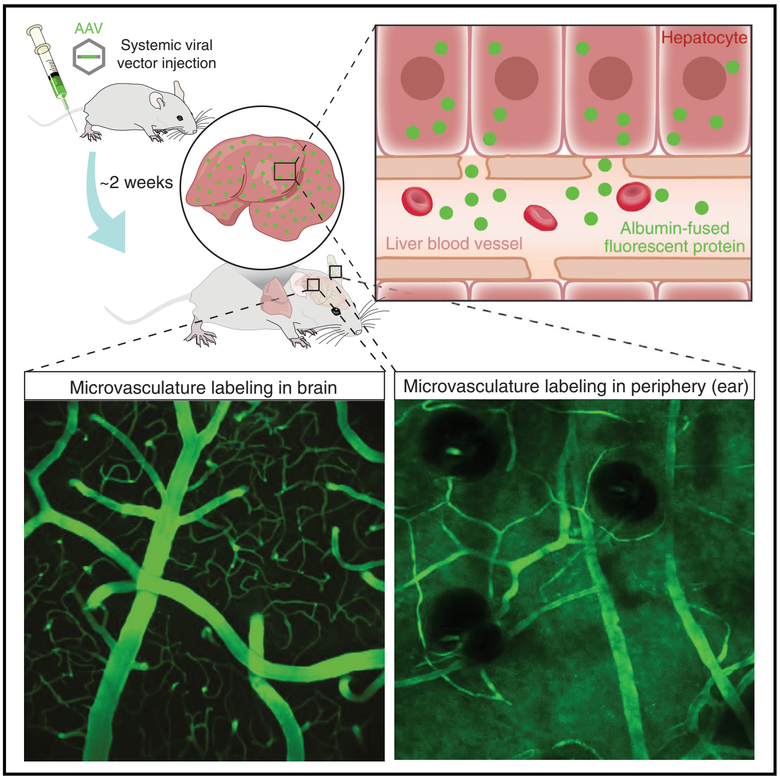

Highlights

- Albumin-fusion fluorescent proteins expressed in the liver are secreted to circulation
- Blood plasma visualization is possible within a week of viral injection
- A single i.p./i.v. viral injection leads to fluorescent blood plasma for several months
- Cerebral and peripheral circulation can be visualized micro- and macroscopically

Authors

Xiaowen Wang, Christine Delle, Antonis Asiminas, ..., Steven A. Goldman, Maiken Nedergaard, Hajime Hirase

Correspondence

maiken_nedergaard@urmc.rochester.edu (M.N.),
hirase@sund.ku.dk (H.H.)

In brief

Our ability to study microcirculation longitudinally is restricted by the short *in vivo* life of injectable fluorescent dyes. Wang et al. describe a method that enables longitudinal visualization of blood plasma in mice after a single injection of AAV viruses, expressing fluorescent secretory albumin-fusion proteins in the liver.

Wang et al., 2022, Cell Reports Methods *2*, 100302
October 24, 2022 © 2022 The Author(s).
https://doi.org/10.1016/j.crmeth.2022.100302

CellPress

Cell Reports Methods

CellPress
OPEN ACCESS

Report

Liver-secreted fluorescent blood plasma markers enable chronic imaging of the microcirculation

Xiaowen Wang,[1,7] Christine Delle,[1,7] Antonis Asiminas,[1,7] Sonam Akther,[1] Marta Vittani,[1] Peter Brøgger,[1] Peter Kusk,[1] Camilla Trang Vo,[1] Tessa Radovanovic,[1] Ayumu Konno,[2,3] Hirokazu Hirai,[2,3] Masahiro Fukuda,[4,5] Pia Weikop,[1] Steven A. Goldman,[1,6] Maiken Nedergaard,[1,6,*] and Hajime Hirase[1,6,8,*]
[1]Center for Translational Neuromedicine, Faculty of Health and Life Sciences, University of Copenhagen, Copenhagen, Denmark
[2]Viral Vector Core, Gunma University Initiative for Advanced Research, Maebashi, Gunma 371-8511, Japan
[3]Department of Neurophysiology & Neural Repair, Gunma University Graduate School of Medicine, Maebashi, Gunma 371-8511, Japan
[4]Program in Neuroscience and Behavioral Disorders, Duke-NUS Medical School, Singapore 169857, Singapore
[5]International Research Center for Medical Sciences (IRCMS), Kumamoto University, Kumamoto, Japan
[6]Center for Translational Neuromedicine, University of Rochester Medical Center, Rochester, NY, USA
[7]These authors contributed equally
[8]Lead contact
*Correspondence: maiken_nedergaard@urmc.rochester.edu (M.N.), hirase@sund.ku.dk (H.H.)
https://doi.org/10.1016/j.crmeth.2022.100302

MOTIVATION Monitoring blood circulation in animal models is critical for understanding many physiological phenomena as well as disease development and progression. Although this is possible with current methods, obtaining longitudinal recordings requires multiple fluorescent tracer injections that can lead to animal stress and alter the physiological relevance of findings. We developed a virus-based method that enables labeling of blood plasma for over months following a single intraperitoneal or intravenous injection.

SUMMARY

Studying blood microcirculation is vital for gaining insights into vascular diseases. Blood flow imaging in deep tissue is currently achieved by acute administration of fluorescent dyes in the blood plasma. This is an invasive process, and the plasma fluorescence decreases within an hour of administration. Here, we report an approach for the longitudinal study of vasculature. Using a single intraperitoneal or intravenous administration of viral vectors, we express fluorescent secretory albumin-fusion proteins in the liver to chronically label the blood circulation in mice. This approach allows for longitudinal observation of circulation from 2 weeks to over 4 months after vector administration. We demonstrate the chronic assessment of vascular functions including functional hyperemia and vascular plasticity in micro- and mesoscopic scales. This genetic plasma labeling approach represents a versatile and cost-effective method for the chronic investigation of vasculature functions across the body in health and disease animal models.

INTRODUCTION

The vertebrate vascular system is an impressive network of vessels providing rapid supply of nutrients and oxygen to tissues and organs throughout the body. The human vascular system reaches a length of nearly 100,000 km, through which the blood circulates within the dense network of capillaries in a matter of a minute (Mescher, 2018). In humans, capillaries have a diameter of 8–10 μm and form the capillary bed in tissues with a density of ~600 1/mm^2, where blood oxygen and metabolites are exchanged (Schmidt, 1989). In mice, cerebral capillaries have a diameter of 4–6 μm (Duelli and Kuschinsky, 1993). Advances in imaging technologies have provided many methods to visualize and study various aspects of circulation and metabolism in animal models. While magnetic resonance imaging and positron emission tomography can capture images of entire body parts, optical imaging provides sufficient temporal and spatial resolutions to characterize the dynamics in individual vessels. In particular, two-photon microscopy provides high lateral spatial resolution (~1 μm) and deep parenchymal penetration (~1 mm). Hence, two-photon imaging has, in recent years, provided a wealth of information regarding the dynamic control of the microcirculation in rodents (Grutzendler and Nedergaard, 2019; Hirase et al., 2004; Kleinfeld et al., 1998; Takano et al., 2006; Wei et al., 2016).

Capillary blood flow in animal models is commonly visualized by introducing a fluorescent tracer into the blood plasma, whereby non-fluorescent blood cells appear dark (Kleinfeld et al., 1998; Seylaz et al., 1999). Fluorescent molecules conjugated to large-fragment dextran (e.g., fluorescein isothiocyanate-dextran [FITC-dextran], 2 MDa) are popular owing to the

This is an open access article under the CC BY license (http://creativecommons.org/licenses/by/4.0/).

absence of immunological response and relatively long plasma lifetime of dextran (Goldenberg et al., 1947; Grönwall and Ingelman, 1945). However, this labeling approach has some important limitations. Introduction of dextran into the blood stream increases its viscosity and consequently reduces flow (Ahn et al., 2020; Dormandy, 1971; Swank and Escobar, 1957). This may have significant implications for the physiological relevance of observations made with dextran blood plasma labeling. Also, the signal intensity of tracer-injected plasma attenuates significantly within an hour of administration, requiring additional intravenous injections for longer experiments. Crucially, awake *in vivo* studies require continuous or repeated tracer administration, which introduces unwanted stress and increases the risk of developing an immune response. A minimally invasive method that allows for stable, long-term monitoring of vascular function will greatly accelerate studies of the microcirculation.

Human and rodent plasma albumin represents ~55% of total plasma protein at concentrations ~0.6 mM (Putnam, 1984; Zaias et al., 2009). The vast majority of plasma albumin (>90%) is synthesized in the liver and rapidly secreted into the bloodstream (Schulze et al., 2019). Therefore, albumin presents a prime candidate for the design of genetically encoded plasma tracer suitable for chronic imaging in animal models. Here, we expressed a secretory recombinant fluorescent protein albumin-mNeonGreen (Alb-mNG) (Shaner et al., 2013) in hepatocytes by intraperitoneal (i.p.) injection of the adeno-associated viral vectors (AAVs) in mice. This genetically encoded tracer was incorporated into the blood plasma, and cerebral capillary flow was reliably observable 2 weeks after virus injection without apparent signs of inflammation. Longitudinal imaging of cerebral capillaries allowed the evaluation of sensory-evoked hyperemia and blood-brain barrier (BBB) permeability in response to lipopolysaccharide-induced inflammation. Overall, here we demonstrate that the visualization of blood flow by liver-secreted albumin-conjugated fluorescent proteins in mice represents a powerful approach to examine acute and chronic changes of vascular structure and function.

RESULTS

Alb-mNG is a secretory protein

We first sought to determine the secretory nature of Alb-mNG. In hepatocytes, secretory mature albumin is derived from preproalbumim, which in turn is processed in the endoplasmic reticulum (ER) and Golgi apparatus to have N terminus cleavages (Schreiber and Urban, 1978). Therefore, we hypothesized that fusion of mNG to the C terminus of albumin (Alb-mNG) will lead to the secretion of Alb-mNG extracellularly, while N terminus fusion (mNG-Alb) should mask the secretory signal and result in cytosolic accumulation. Accordingly, we transfected HEK293 cells with mammalian expression plasmids containing the Alb-mNG and mNG-Alb constructs (Figures 1A and 1B). A secretory form of mNG containing the immunoglobulin K (IgK) leader signal peptide at the N terminus (IgKL-mNG) was used as a positive control. HEK293T cells were imaged 1 and 2 days after transfection. Green fluorescence was detected in transfected cells with each of the three plasmids, and no apparent signs of abnormal morphology were observed.

As expected, IgKL-mNG and Alb-mNG expression resulted in dim cytosolic fluorescent signals and obvious culture medium fluorescence (two-way ANOVA: construct × time interaction, $p < 0.05$; *post hoc* tests: IgKL-mNG versus mNG-Alb and Alb-mNG versus mNG-Alb, $p < 0.05$ at both 24 and 48 h time points; n = 18; Figures 1C–1E). Non-uniform intracellular localization was evident, possibly indicating intracellular trafficking or lysosomal processing. On the other hand, bright cytosolic expression was observed for mNG-Alb (Figure 1C). Secretion of IgKL-mNG and Alb-mNG was further confirmed by measurement of culture medium fluorescence using a plate reader 24 and 48 h post-transfection (two-way ANOVA: construct × time interaction, $p < 0.05$; *post hoc* tests: IgKL-mNG versus mNG-Alb and Alb-mNG versus mNG-Alb, $p < 0.05$ at both 24 and 48 h time points; n = 6; Figure 1E). Notably, medium fluorescence increased over time, suggesting stability and accumulation of the secreted proteins. These observations indicated that C terminus fusion of albumin to fluorescent proteins can function as a genetically encoded secretory tracer. In particular, when expressed in liver hepatocytes *in vivo*, albumin-fused fluorescent tracers should be incorporated into the blood via large fenestrated hepatic capillaries.

Plasma is robustly and chronically visualized by *in vivo* hepatocyte transgene expression

To achieve *in vivo* expression of Alb-mNG in the liver, an AAV serotype 8 (AAV8) was utilized owing to its high affinity to hepatocytes (Aronson et al., 2019; Cunningham et al., 2008; Sands, 2011; Zincarelli et al., 2008). The minimal transthyretin promoter P3 was used to achieve hepatocyte-specific expression (Figure 2A) (Nair et al., 2014; Viecelli et al., 2014). We confirmed strong hepatocytic expression of the AAV by systemic injection of AAV8-P3-EGFP (intravenous [i.v.], 2×10^{11} vg, 3 weeks post-injection; Figure 2D). Liver exhibited high EGFP expression in virtually all hepatocytes (Figure 2D) in agreement with previous reports (Malato et al., 2011). We find that the fluorescence signal of AAV8-P3-Alb-mNG-infected liver was relatively mild, most likely due to the secretory nature of Alb-mNG (Figure 2D).

We next examined the presence of Alb-mNG in blood plasma. Blood samples were collected over an 8 week period after AAV8 administration (Figures 2A and 2B). Examination of blood samples in glass micropipettes showed that the fluorescence signal can be detected as early as 2 days after injection and become brighter on the 5th day. Longitudinal quantification of plasma fluorescence showed that the signal peaks at 3–4 weeks post-injection and the expression lasts over 8 weeks (one-way ANOVA: significant effect of time, $p < 0.05$; n = 6 mice; Figure 2C). Plasma concentrations of mNG and albumin were further quantified during the 8 week time frame (Figures 2E and 2F). Quantitative fluorescence measurement using known concentrations of mNG as reference shows a similar temporal profile of mNG plasma concentration to our glass-pipette assay, generally indicating ~1 μM plasma alb-mNG at 2 weeks or after post-injection (one-way ANOVA: no significant effect of time, $p > 0.05$; n = 3 mice; Figure 2E). Moreover, total plasma albumin concentration (endogenous and Alb-mNG) was stable (two-way ANOVA: no significant effect of time, treatment or interaction, $p > 0.05$; n = 3–4 mice; Figure 2F) and within the range of the published murine serum albumin concentration (20–30 mg/mL = 300–450 μM; Figure 2F),

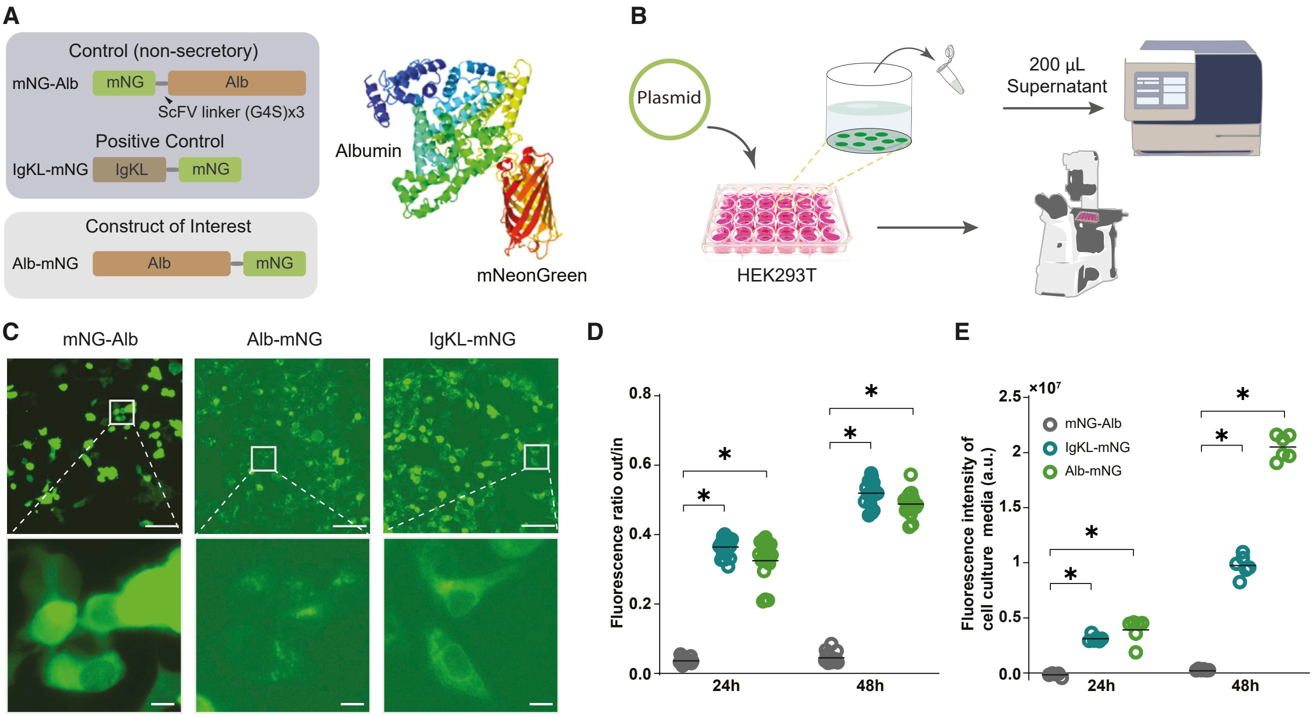

Figure 1. Construction and validation of secretory fluorescent protein-tagged albumin

(A) Schematic construct design of mNG-Alb (non-secretory negative control), IgkL-mNG (secretory positive control), and Alb-mNG. A three-dimensional (3D) protein structure prediction for Alb-mNG is displayed on the right.

(B) Schematic illustration for cell culture testing of the plasmid constructs.

(C) Microscopic images of transfected HEK293T cells at 48 h for mNG-Alb, Alb-mNG, and IgKL-mNG. The bottom row is the magnification of the corresponding white squares in the top row. Scale bars, top: 100 μm, bottom: 10 μm.

(D) Fluorescence signal ratio of external versus cytosolic signal of microscopic images taken at 24 and 48 h. n = 18.

(E) Fluorescence intensity of cell culture medium from 24 to 48 h post-transfection measured via a microplate reader. n = 6.

All graphs show mean (line) and individual values; *p < 0.05.

suggesting normal albumin-oriented osmotic homeostasis (Zaias et al., 2009).

Alb-mNG does not lead to inflammation *in vivo* or abnormal spontaneous behavior

To examine possible immune responses due the recombinant protein expression, we measured plasma C-reactive protein (CRP) levels, a standard systemic marker for tissue inflammation (Liang et al., 2019; Pepys and Hirschfield, 2003). As a result, plasma CRP levels in AAV-injected mice were comparable to those of saline-injected controls (during 8 weeks post-AAV injection; t test $p > 0.05$; n [control] = 6, n [Alb-mNG] = 12 mice; Figure 2G). To further assess possible tissue inflammation, we visualized liver macrophages and brain microglia by IBA1 immunohistochemistry (Figure 2H). Accordingly, the morphology of liver macrophages and brain microglia did not reveal any signs of inflammation in AAV8-injected mice (Figure 2H). Moreover, neither body weight nor open-field ambulatory behavior were impacted by AAV8-P3-Alb-mNG or AAV8-P3-IgKL-mNG 4 weeks post-administration (Figure S1) (Burkholder et al., 2012). Taken together, these experiments demonstrate the minimal footprint of our plasma labeling approach on host physiology and behavior.

Alb-mNG is superior to fluorescent-conjugated dextran for chronic study of circulation and vasculature

Having confirmed the plasma fluorescence by a single i.p. AAV injection, we imaged the cerebral vasculature of AAV-injected mice through a chronic cranial window (Figure 3A). Consistent with the plasma measurements, blood plasma was visualized by two-photon microscopy for at least 8 weeks after AAV injection. To compare Alb-mNG fluorescence with acute administration fluorescent dyes, we administered Texas Red dextran (70 kDa) to mice expressing Alb-mNG (Figures 3A and 3B). The labeled dextran showed a perfect match to the vascular pattern visualized by Alb-mNG 10 min after administration. However, the signal intensity dropped substantially during the first hour of imaging. By contrast, Alb-mNG yielded a stable signal during the 2 h recording session (two-way ANOVA: probe × time interaction, $p < 0.05$; *post hoc* tests: mNG-Alb versus Texas Red, $p < 0.05$ at both 1 and 2 h time points; n = 3; Figure 3C). To further evaluate the utility of Alb-mNG for the longitudinal monitoring of vasculature, the cortical microvasculature was imaged at 3 and 7 weeks after AAV administration. While the vast majority of the microvasculature remained structurally similar across the imaging sessions, a few examples of vascular plasticity were noted (Figures 3D and 3E). The robust visualization of blood plasma

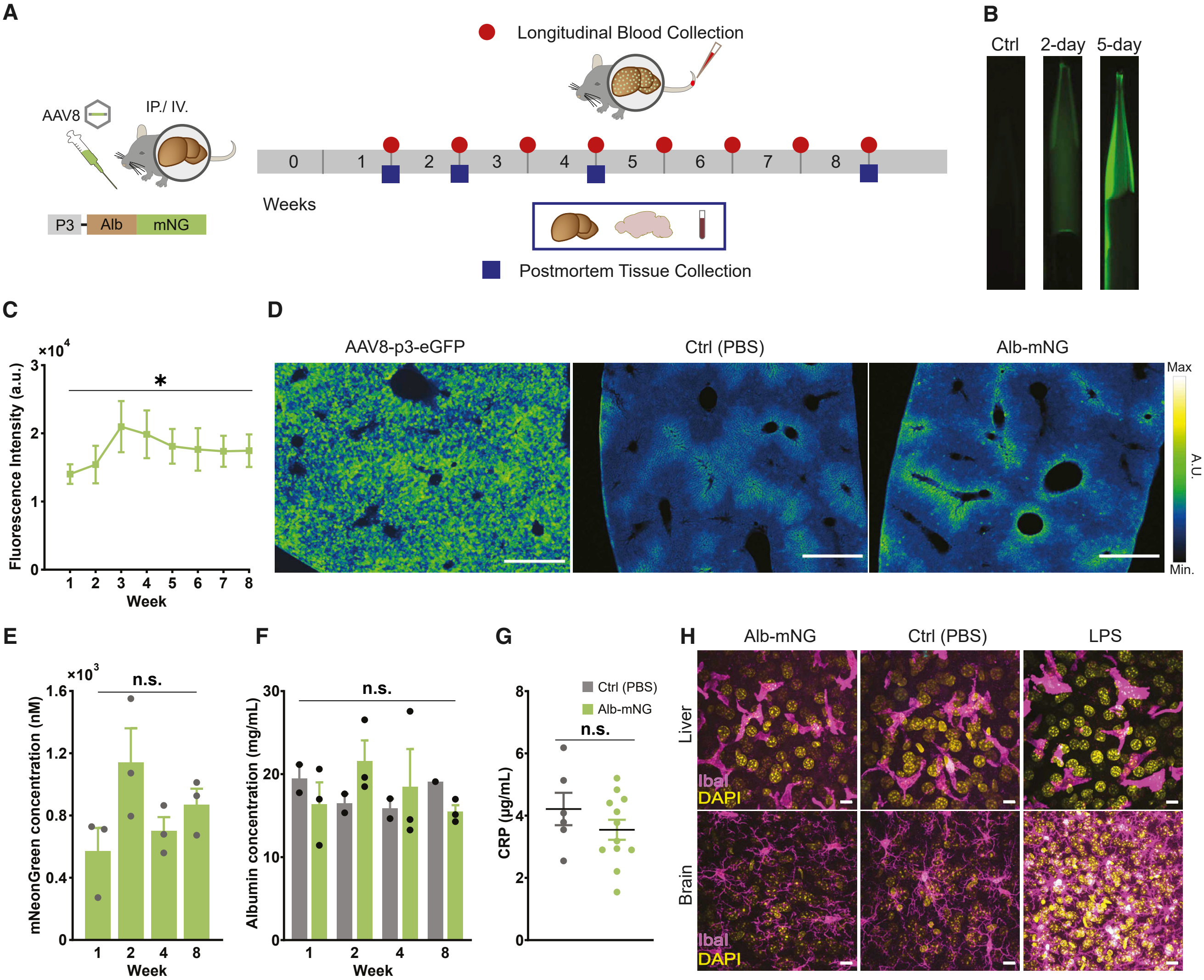

Figure 2. Robust and chronic visualization of blood plasma by *in vivo* transgene expression of Alb-mNG in hepatocytes

(A) Schematic of approach for the *in vivo* experiments. AAV8-P3-Alb-mNG is administrated to mice via i.p. or i.v. injection (left). Alb-mNG expression was monitored by collecting a blood sample from the tail. Brain and liver tissues and blood were collected for morphological and biochemical examination (right).

(B) Example of the fluorescence signals in blood samples collected on days 2 and 5 from a mouse that received i.p injection of AAV8-P3-Alb-mNG.

(C) Plasma Alb-mNG fluorescence intensity over a time course of 8 weeks. Significant effect of time (one-way ANOVA: $p < 0.05$) (n = 6 mice).

(D) Mouse liver images after 3 weeks of AAV8-P3-eGFP (positive control), PBS (negative control), and AAV8-P3-Alb-mNG i.p. injection. Scale bars, 500 μm.

(E) mNG concentration in plasma samples (n = 3 mice). No significant effect of time (one-way ANOVA: $p > 0.05$)

(F) Plasma albumin concentration using albumin ELISA in PBS- (gray, n = 2) and Alb-mNG-injected (green, n = 3) mice.

(G) Plasma CRP levels for control or Alb-mNG i.p. injected mice during the 8 weeks of post-injection period (n = 6–12 mice).

(H) Example images of liver (top panel) and brain slices (bottom panel) of control- (PBS) or Alb-mNG-expressing mice immunostained for macrophages (liver) or microglia (brain) by IBA1 (purple) and DAPI (yellow). Brain sections of lipopolysaccharide (LPS)-injected mice displayed reactive microglia morphology, while resting microglia are observed in the Alb-mNG mouse. Scale bars, 10 μm.

Graphs show means ± SEM and individual values (except from C); *p < 0.05.

allows for the longitudinal study of microcirculation. To this end, we conducted high frame rate imaging (160–220 Hz) on selected areas containing a single capillary. We demonstrate that the expression of the albumin-fusion tracer reaches the levels required for this fast imaging regime at 3 weeks post-AAV injection. Indeed, captured capillary images show clear black and white stripes where the black areas indicate the presence of red blood cells (RBCs) 3 and 7 weeks after AAV injection in the same animal (Figures 3F and 3G, left panels). Travel time between two points in a capillary was estimated by calculating the cross-correlogram of the time-signal intensity data (Figures 3F and 3G, right panels), hence mean flow speeds of 1.1 and 5.2 mm/s were computed for the two example captures (Chaigneau et al., 2003). These values were within the previously

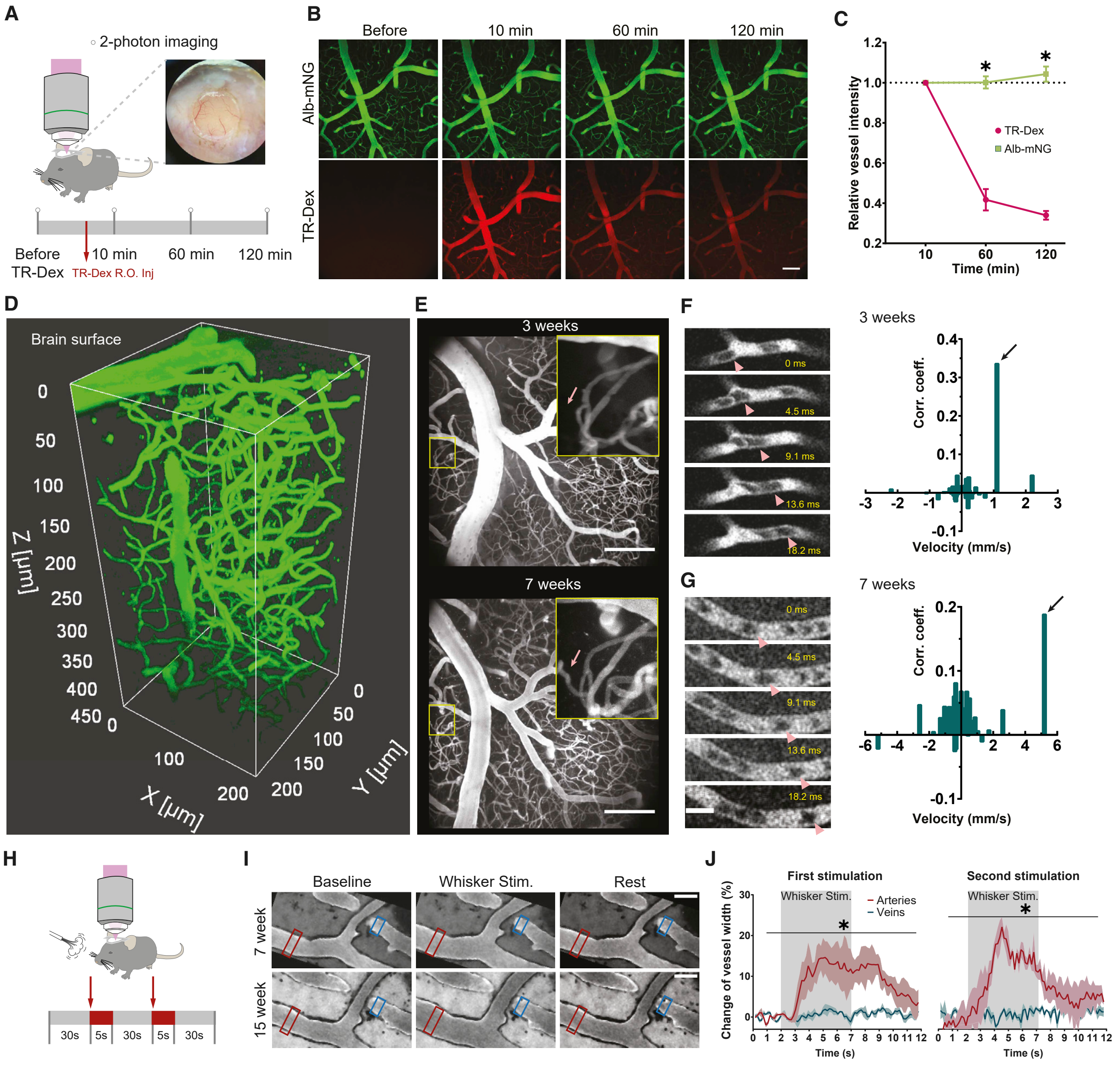

Figure 3. Genetic expression of Alb-mNG is advantageous to fluorescent dextran in long-lasting imaging sessions

(A) Experimental approach to compare the genetically expressed Alb-mNG with i.v.-injected Texas Red dextran (70 kDa). Alb-mNG-expressing mice (7–8 weeks) were imaged under ketamine-xylazine anesthesia before and 10, 60, and 120 min after Texas Red dextran injection.

(B) Example images at various time points during an imaging session. Alb-mNG signal is present throughout the total imaging session with little attenuation. Texas Red dextran signals diminishes within an hour. Scale bar, 100 µm.

(C) Quantification of signal intensity for Alb-mNG and Texas Red dextran for the time course of 120 min (n = 3). Graph shows means ± SEM; *post hoc* between Alb-mNG and Texas Red, *$p < 0.05$.

(D) Volumetric imaging of brain vasculature covering 450 µm below the pial surface of Alb-mNG-expressing mouse (post-injection 10 weeks).

(E) Two-photon images obtained from the same mouse at 3 and 7 weeks of Alb-mNG expression. The zoomed in area (yellow square) depicts neovascularization at 7 weeks (red arrow). Scale bar, 100 µm.

(F and G) Two-photon imaging of a capillary at 3 and 7 weeks of Alb-mNG expression at frame rate of 116–220 Hz enables quantification of blood flow velocity by computing the cross-correlogram (right). See histogram on the right side. Pink triangles indicate the flow of an example red blood cell. Scale bar, 10 µm.

(H) Experimental setup for functional hyperemia induced by whisker puff. During each session, mice were exposed to two whisker stimulations. Each session started with a 30 s baseline (gray), followed by 5 s of whisker stimulation (red) (50-ms pulses at 10 Hz). After the second stimulation, recording continued for 30 s (gray).

(legend continued on next page)

reported mean flow speed range (Chaigneau et al., 2003; Unekawa et al., 2010). To further evaluate the utility of liver-secreted plasma tracers, we imaged the microvasculature in the whisker-barrel cortex while stimulating whiskers. We demonstrate that functional hyperemia can be induced in the plasma-labeled mice by whisker stimulation, as previously shown (Figure 3H) (Rungta et al., 2021; Takata et al., 2013). Our method allows chronic assessment of functional hyperemia for more than 7 weeks after AAV administration (two-way ANOVA: vessel type × time interaction, $p < 0.05$; at both first and second whisker stimulation; n [arteries] = 3, n [veins] = 3 from 3 mice; Figures 3I and 3J).

To address the importance of the molecular size of liver-secreted plasma fluorescent tracer, we also expressed IgKL-mNG in the liver using the same AAV8 approach (Figure S2). Long-term visualization of blood plasma was also possible with this viral construct; however, plasma fluorescent intensity was an order of magnitude lower than that of Alb-mNG (Figures 2B and 2D), pointing toward the importance of the molecular size for vascular leakage. Quantification of total albumin revealed similar concentrations as controls and Alb-mNG (Figure S2C), with no signs of systemic inflammation (Figures S2E and S2F). Despite the decreased plasma fluorescence compared with Alb-mNG, imaging of cerebral vasculature was feasible (Figure S2G), including fast capillary imaging for RBC flow (Figure S2H).

To extend the toolbox of Alb-fused proteins as chronic plasma tracers, we designed a different vector by substituting mNG with a bright red fluorescent protein mScarlet (Alb-mScarlet) (Bindels et al., 2017). As with Alb-mNG, systemic administration of AAV8-P3-Alb-mScarlet (i.v., via the retro-orbital sinus) achieved robust long-term plasma visualization by two-photon microscopy. Given that both i.v. and i.p. administration of AAV lead to successful blood plasma labeling, we compared it between the two administration routes. We observed no apparent effects of administration route on plasma fluorescence intensity (Figure S3C). Furthermore, we have also successfully generated albumin-fused tracers with Rosmarinus (cyan) (Molina et al., 2017) and mCarmine (deep red) (Fabritius et al., 2018), which are available via Addgene (see STAR Methods; Figure S3).

Besides cerebral vasculature, long-term monitoring of vasculature in peripheral tissue is also possible. We demonstrate this by imaging vasculature in the ear (Honkura et al., 2018). Even at 10 weeks after a single i.p. injection of Alb-mNG viral construct, the fluorescent signal is strong and sufficient even for fast imaging of capillary RBC flow in the periphery (Figure S4).

We next assessed whether our approach can be used in combination with other virally delivered tools. We have previously shown that AAV administration leads to the generation of neutralizing antibodies that limit the infection of AAVs inoculated after a few days or later (Shinohara et al., 2019). Therefore, we performed brain parenchymal injections of AAV9 and AAV8 in mice followed by retro-orbital administration (i.v.) of AAV8-P3-Alb-mScarlet either 12 weeks after or within 1 h. Plasma fluorescence was only evident in mice that received all viral vectors within 1 h (Figure S5).

Finally, we tested whether our fluorescent tracers are adequate for macroscopic study of cerebral vasculature. Major vessels on the brain surface can be readily imaged through a cranial window using a wide-field fluorescent microscope 4 weeks after AAV administration of both Alb-mScarlet and Alb-mNG (Figure 4A) (Ahn et al., 2020). Notably though, the image contrast between vessels and parenchymal background is enhanced in Alb-mScarlet, resulting in a significantly higher signal-to-noise ratio of vascular imaging (signal/background ratio: Mann Whitney: $p < 0.05$; Shannon's entropy: Mann Whitney: $p < 0.05$; n [Alb-mNG] = 10, n [Alb-mScarlet] = 6; Figures 4B and 4C).

DISCUSSION

Chronic monitoring of the vasculature is crucial for the study of developmental processes (Coelho-Santos et al., 2021; Mukouyama et al., 2002) and aging (Ungvari et al., 2018) as well as disease progression (Montagne et al., 2021), recovery (Williamson et al., 2020), and evaluation of therapeutic effects in animal models. Currently, imaging of the vasculature in mice requires repeated i.v. injection of dextran-conjugated fluorescent dyes. We present an innovative genetic approach that enables robust labeling of plasma for more than 3 months. A single i.p. or i.v. injection of AAV induces hepatocyte expression of fluorescent protein-tagged albumin and hence achieves labeling of blood plasma. No additional manipulations are required, making this approach ideal for the study of both wild-type and genetically modified mice. We present four implementations of this approach using the monomeric fluorescent proteins mNeonGreen (Alb-mNG), mScarlet (Alb-mScarlet), mCarmine (Alb-mCarmine), and Rosmarinus (Alb-Rosmarinus) and demonstrate that fluorescent protein-tagged albumin is superior to dextran-conjugated fluorescent dyes. A key strength is the minimally invasive and long-lasting nature of this approach. While our monitoring period was limited by our animal experimentation license, previous studies report sustained AAV-mediated gene expression for over 9 months (Zincarelli et al., 2008). This suggests that the AAV-mediated plasma probes can be used to track the vasculature for a significant portion of the rodent lifespan.

Previously, Xie and colleagues reported the generation of a transgenic zebrafish model expressing vitamin D-binding protein (DBP)-EGFP in the liver that results in blood plasma labeling (Xie et al., 2010). While that approach is similar to our liver-expressed, plasma-secreted fluorescent probes, our AAV-mediated approach offers several advantages. It eliminates the need for breeding transgenic lines, offers great flexibility with regards to the nature and properties of the albumin-fused recombinant protein, and allows for better control of expression levels by modulating the concentration of viral injections.

(I) Example two-photon images of the same mouse at 7 and 15 weeks expression of Alb-mNG show dilation of artery (marked in red square) compared with vein (marked in blue square) after air puff whisker stimulation. Scale bar 50 μm.

(J) Quantification of percentage change of vessel width for artery and vein (n =3 for each vessel type) after two whisker stimulations. Graphs show means ± SEM; Vessel type × time interaction, *$p < 0.05$.

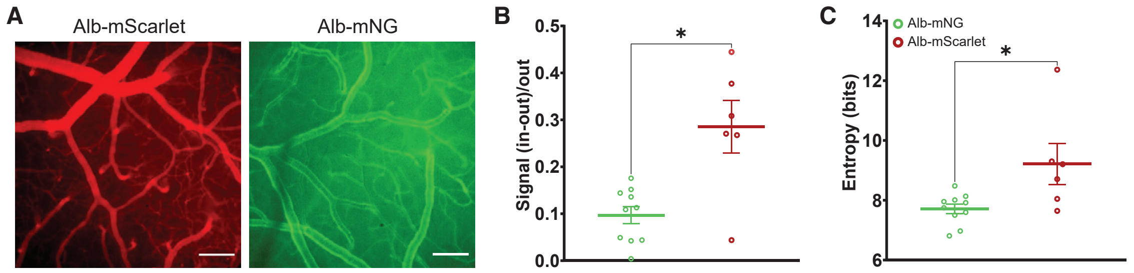

Figure 4. Comparison between Alb-mNG and Alb-mScarlet macroscopic fluorescent imaging
(A) Representative examples of macroscopic imaging with the two fluorescent plasma probes. Scale bars, 50 μm.
(B) Signal-to-noise ratio quantification.
(C) Shannon's entropy of macroscopic images.
All graphs show means ± SEM and individual values; *$p < 0.05$. $N_{Alb\text{-}mNG} = 10$, $N_{Alb\text{-}mScarlet} = 6$.

A crucial advantage of our approach is the ease by which one can collect more physiologically relevant data compared with acutely injected tracers such as fluorescent dextran. Our genetic approach diminishes the induced stress and complications from repeated i.v. injections, especially when performed on awake mice. Moreover, the expression of albumin-fusion probes avoids the documented concern about blood viscosity increase by dextran infusion (Goldenberg et al., 1947; Grönwall and Ingelman, 1945). While we did not directly measure viscosity in blood samples, our quantification of recombinant and total albumin in the plasma suggests that viscosity change by the current protocol is unlikely since the total albumin level remains virtually unchanged for the observation period of several months (Figure 2F). Moreover, no signs of systemic or tissue inflammation were noted as assessed by histology and the CRP assay. In addition, unaltered open-field activity and body weight strongly supports the suitability of the plasma probe for chronic experiments. Of note, the recombinant albumin is derived from the murine albumin sequence with an intention to minimize immune reactivity in mice.

Undoubtedly, the most exciting application of genetically encoded plasma visualization is the longitudinal study of vascular circulation. Coupling with functional imaging of distinct cell types such as endothelial cells, pericytes, or astrocytes is expected to provide critical insights into various processes pertinent to circulation including angiogenesis and vascular plasticity (Iadecola and Nedergaard, 2007; Nikolakopoulou et al., 2019; Payne et al., 2018). The technique accommodates experimental designs that span several months with lasting plasma signal intensity (Figures 3B and 3C). The high signal-to-noise ratio and long-term expression achieved by the current method should enable daily assessment of changes of blood circulation and BBB permeability.

For wide-field imaging, Alb-mScarlet has a clear advantage over Alb-mNG due to the low intrinsic fluorescence of the brain tissue in the red spectra (Bindels et al., 2017). The long-lasting labeling of blood plasma combined with the large field of view of wide-field macroscopes offers a great opportunity to assess vascular dynamics and function over large cortical regions, longitudinally in health and disease. These studies allow for crucial within-subject comparisons that will enhance statistical power and aid ongoing efforts to reduce animal usage in biomedical research.

We find that i.p. injection yields reliable plasma probe expression, likely reflecting that the primary route of AAV particle absorption is through the mesenteric vessels, which drain into the portal vein of the liver (Lukas et al., 1971). If this is the case, i.p.-injected AAV particles reach hepatocytes before entering systemic circulation. While lower quantities of viral constructs are needed for retro-orbital injections ($^1/_3$ of i.p.), i.p. injection offers a few advantages including a simpler procedure, shorter administration time, adaptability to awake animals, and higher reproducibility (Turner et al., 2011). The low biosafety level of AAV usage makes it possible to use this method in all standard laboratories. Moreover, the prevalence of AAV technology has made this technique financially affordable (e.g., Addgene or VVF Zurich).

Although in the current work we demonstrate the utility of our fluorescent protein-tagged albumin constructs only in mice, our approach should benefit larger laboratory animal models as well. Based on the liver volume of regularly used animal species and technical limitations to virus production, we expect a single viral i.v. or i.p. injection to be feasible up to the size of a mini pig, which has a liver size of approximately 100 times that of an adult mouse (Elefson et al., 2021; Hall et al., 2012).

Here, we present four implementations of liver-secreted albumin-fusion proteins. The ever-growing toolbox of optical biosensors and optical manipulation tools, as well as advances in miniaturized microscopy, provide huge opportunities for the longitudinal study of circulation in a near-physiological, naturalistic manner. Coupled with rodent disease models, liver-secreted biosensors and other genetically encoded tools open the way for exploring causal relationships between circulation and disease pathophysiology.

Limitations of the study

While our approach can be combined with other genetic manipulations (e.g., genetically modified animals, other viral injections), we have shown that repeated injections do not work, most likely due to the generation of neutralizing antibodies that limit the infection of AAVs (Figure S5). This limitation should be taken into account when designing experiments that require the administration of multiple AAVs, including microinjections into the brain parenchyma.

The four albumin-fused, liver-secreted fluorescent probes we present here (Alb-mNG, mScarlet, Alb-mCarmine, and Alb-Rosmarinus) are just a small sample of the vast number of possible implementations of our approach. However, the packing capacity of AAVs (~4.7kb) limits the size of fluorescent probes and markers that can be used.

STAR★METHODS

Detailed methods are provided in the online version of this paper and include the following:

- KEY RESOURCES TABLE
- RESOURCE AVAILABILITY
  - Lead contact
  - Materials availability
  - Data and code availability
- EXPERIMENTAL MODEL AND SUBJECT DETAILS
- METHOD DETAILS
  - DNA constructs
  - Cell culture
  - *In vivo* recombinant protein expression
  - Biochemical analysis
  - Open field test
  - Histology
  - *In vivo* fluorescence imaging
- QUANTIFICATION AND STATISTICAL ANALYSIS
  - Image data analysis
  - Statistical analysis

SUPPLEMENTAL INFORMATION

Supplemental information can be found online at https://doi.org/10.1016/j.crmeth.2022.100302.

ACKNOWLEDGMENTS

We thank Dr. Jean-Charles Paterna (University of Zurich Viral Vector Facility) for technical support on AAV vectors. We also thank members of the Center for Translational Neuromedicine for comments on earlier versions of the manuscript and Nathalie Jurisch-Yaksi (NTNU) for providing helpful feedback. We thank Dan Xue for the graphical abstract. This work was supported by the Novo Nordisk Foundation (H. Hirase, NNF19OC0058058), Independent Research Fund Denmark (H. Hirase, 0134-00107B), the Olav Thon Foundation (M.N.), the Adelson Foundation (M.N.), NIH-NINDS Brain Initiative Award (U19NS128613, H. Hirase and M.N.), the program for Brain Mapping by Integrated Neurotechnologies for Disease Studies (Brain/MINDS, JP20dm0207057 and JP21dm0207111) (H. Hirai), Japan Agency for Medical Research and Development (AMED JP21dm0207111) (H. Hirai), and JSPS KAKENHI, MEXT, Japan (19K06899 and 22K06454) (A.K.).

AUTHOR CONTRIBUTIONS

Designed research, X.W., C.D., A.A., S.A., M.F., A.K., S.A.G., M.N., and H. Hirase; performed research, X.W., C.D., S.A., C.V.T., M.V., P.B., P.K., A.K., and T.R.; contributed unpublished reagents/analytic tools, A.K. and H. Hirai; analyzed/interpreted data, X.W., C.D., A.A., S.A., and C.V.T.; wrote the paper, A.A., C.D., X.W., M.F., M.N., and H. Hirase.

DECLARATION OF INTERESTS

The authors declare no competing interests.

Received: April 7, 2022
Revised: August 11, 2022
Accepted: September 1, 2022
Published: September 21, 2022

REFERENCES

Ahn, S.J., Ruiz-Uribe, N.E., Li, B., Porter, J., Sakadzic, S., and Schaffer, C.B. (2020). Label-free assessment of hemodynamics in individual cortical brain vessels using third harmonic generation microscopy. Biomed. Opt Express *11*, 2665–2678. https://doi.org/10.1364/BOE.385848.

Aronson, S.J., Bakker, R.S., Shi, X., Duijst, S., ten Bloemendaal, L., de Waart, D.R., Verheij, J., Ronzitti, G., Oude Elferink, R.P., Beuers, U., et al. (2019). Liver-directed gene therapy results in long-term correction of progressive familial intrahepatic cholestasis type 3 in mice. J. Hepatol. *71*, 153–162. https://doi.org/10.1016/j.jhep.2019.03.021.

Bindels, D.S., Haarbosch, L., van Weeren, L., Postma, M., Wiese, K.E., Mastop, M., Aumonier, S., Gotthard, G., Royant, A., Hink, M.A., et al. (2017). mScarlet: a bright monomeric red fluorescent protein for cellular imaging. Nat. Methods *14*, 53–56. https://doi.org/10.1038/nmeth.4074.

Burkholder, T., Foltz, C., Karlsson, E., Linton, C.G., and Smith, J.M. (2012). Health evaluation of experimental laboratory mice. Curr. Protoc. Mouse Biol. *2*, 145–165. https://doi.org/10.1002/9780470942390.mo110217.

Chaigneau, E., Oheim, M., Audinat, E., and Charpak, S. (2003). Two-photon imaging of capillary blood flow in olfactory bulb glomeruli. Proc. Natl. Acad. Sci. USA *100*, 13081–13086. https://doi.org/10.1073/pnas.2133652100.

Coelho-Santos, V., Berthiaume, A.-A., Ornelas, S., Stuhlmann, H., and Shih, A.Y. (2021). Imaging the construction of capillary networks in the neonatal mouse brain. Proc. Natl. Acad. Sci. USA *118*, e2100866118. https://doi.org/10.1073/pnas.2100866118.

Cunningham, S.C., Dane, A.P., Spinoulas, A., and Alexander, I.E. (2008). Gene delivery to the juvenile mouse liver using AAV2/8 vectors. Mol. Ther. *16*, 1081–1088. https://doi.org/10.1038/mt.2008.72.

Dormandy, J.A. (1971). Influence of blood viscosity on blood flow and the effect of low molecular weight dextran. Br. Med. J. *4*, 716–719. https://doi.org/10.1136/bmj.4.5789.716.

Duelli, R., and Kuschinsky, W. (1993). Changes in brain capillary diameter during hypocapnia and hypercapnia. J. Cerebr. Blood Flow Metabol. *13*, 1025–1028. https://doi.org/10.1038/jcbfm.1993.129.

Elefson, S.K., Lu, N., Chevalier, T., Dierking, S., Wang, D., Monegue, H.J., Matthews, J.C., Jang, Y.D., Chen, J., Rentfrow, G.K., et al. (2021). Assessment of visceral organ growth in pigs from birth through 150 kg. J. Anim. Sci. *99*. https://doi.org/10.1093/jas/skab249.

Fabritius, A., Ng, D., Kist, A.M., Erdogan, M., Portugues, R., and Griesbeck, O. (2018). Imaging-based screening platform assists protein engineering. Cell Chem. Biol. *25*, 1554–1561.e8. https://doi.org/10.1016/j.chembiol.2018.08.008.

Goldenberg, M., Crane, R.D., and Popper, H. (1947). Effect of intravenous administration of dextran, a macromolecular carbohydrate, in animals. Am. J. Clin. Pathol. *17*, 939–948. https://doi.org/10.1093/ajcp/17.12.939.

Grönwall, A., and Ingelman, B. (1945). Dextran as a substitute for plasma. Nature *155*, 45. https://doi.org/10.1038/155045a0.

Grutzendler, J., and Nedergaard, M. (2019). Cellular control of brain capillary blood flow: in vivo imaging veritas. Trends Neurosci. *42*, 528–536. https://doi.org/10.1016/j.tins.2019.05.009.

Hall, C., Lueshen, E., Mošat', A., and Linninger, A.A. (2012). Interspecies scaling in pharmacokinetics: a novel whole-body physiologically based modeling framework to discover drug biodistribution Mechanisms in vivo. J Pharm. Sci. *101*, 1221–1241. https://doi.org/10.1002/jps.22811.

Hirase, H., Creso, J., and Buzsáki, G. (2004). Capillary level imaging of local cerebral blood flow in bicuculline-induced epileptic foci. Neuroscience *128*, 209–216. https://doi.org/10.1016/j.neuroscience.2004.07.002.

Honkura, N., Richards, M., Laviña, B., Sáinz-Jaspeado, M., Betsholtz, C., and Claesson-Welsh, L. (2018). Intravital imaging-based analysis tools for vessel

identification and assessment of concurrent dynamic vascular events. Nat. Commun. *9*, 2746. https://doi.org/10.1038/s41467-018-04929-8.

Iadecola, C., and Nedergaard, M. (2007). Glial regulation of the cerebral microvasculature. Nat. Neurosci. *10*, 1369–1376. https://doi.org/10.1038/nn2003.

Kelley, L.A., Mezulis, S., Yates, C.M., Wass, M.N., and Sternberg, M.J.E. (2015). The Phyre2 web portal for protein modeling, prediction and analysis. Nat. Protoc. *10*, 845–858. https://doi.org/10.1038/nprot.2015.053.

Kleinfeld, D., Mitra, P.P., Helmchen, F., and Denk, W. (1998). Fluctuations and stimulus-induced changes in blood flow observed in individual capillaries in layers 2 through 4 of rat neocortex. Proc. Natl. Acad. Sci. USA *95*, 15741–15746. https://doi.org/10.1073/pnas.95.26.15741.

Konno, A., and Hirai, H. (2020). Efficient whole brain transduction by systemic infusion of minimally purified AAV-PHP. eB. J Neurosci Methods *346*, 108914. https://doi.org/10.1016/j.jneumeth.2020.108914.

Liang, C., Li, J., Lu, C., Xie, D., Liu, J., Zhong, C., Wu, X., Dai, R., Zhang, H., Guan, D., et al. (2019). HIF1α inhibition facilitates Leflunomide-AHR-CRP signaling to attenuate bone erosion in CRP-aberrant rheumatoid arthritis. Nat. Commun. *10*, 4579. https://doi.org/10.1038/s41467-019-12163-z.

Lukas, G., Brindle, S.D., and Greengard, P. (1971). The route of absorption of intraperitoneally administered compounds. J. Pharmacol. Exp. Therapeut. *178*. LP – 566.

Malato, Y., Naqvi, S., Schürmann, N., Ng, R., Wang, B., Zape, J., Kay, M.A., Grimm, D., and Willenbring, H. (2011). Fate tracing of mature hepatocytes in mouse liver homeostasis and regeneration. J. Clin. Invest. *121*, 4850–4860. https://doi.org/10.1172/JCI59261.

Mescher, A.L. (2018). The circulatory system. Junqueira's Basic Histology: Text and Atlas, *15e* (McGraw-Hill Education)).

Molina, R.S., Tran, T.M., Campbell, R.E., Lambert, G.G., Salih, A., Shaner, N.C., Hughes, T.E., and Drobizhev, M. (2017). Blue-shifted green fluorescent protein homologues are brighter than enhanced green fluorescent protein under two-photon excitation. J. Phys. Chem. Lett. *8*, 2548–2554. https://doi.org/10.1021/acs.jpclett.7b00960.

Montagne, A., Nikolakopoulou, A.M., Huuskonen, M.T., Sagare, A.P., Lawson, E.J., Lazic, D., Rege, S. v, Grond, A., Zuniga, E., Barnes, S.R., et al. (2021). APOE4 accelerates advanced-stage vascular and neurodegenerative disorder in old Alzheimer's mice via cyclophilin A independently of amyloid-β. Nat. Aging *1*, 506–520. https://doi.org/10.1038/s43587-021-00073-z.

Mukouyama, Y., Shin, D., Britsch, S., Taniguchi, M., and Anderson, D.J. (2002). Sensory nerves determine the pattern of arterial differentiation and blood vessel branching in the skin. Cell *109*, 693–705. https://doi.org/10.1016/S0092-8674(02)00757-2.

Nair, N., Rincon, M.Y., Evens, H., Sarcar, S., Dastidar, S., Samara-Kuko, E., Ghandeharian, O., Man Viecelli, H., Thöny, B., de Bleser, P., et al. (2014). Computationally designed liver-specific transcriptional modules and hyperactive factor IX improve hepatic gene therapy. Blood *123*, 3195–3199. https://doi.org/10.1182/blood-2013-10-534032.

Nikolakopoulou, A.M., Montagne, A., Kisler, K., Dai, Z., Wang, Y., Huuskonen, M.T., Sagare, A.P., Lazic, D., Sweeney, M.D., Kong, P., et al. (2019). Pericyte loss leads to circulatory failure and pleiotrophin depletion causing neuron loss. Nat. Neurosci. *22*, 1089–1098. https://doi.org/10.1038/s41593-019-0434-z.

Oe, Y., Wang, X., Patriarchi, T., Konno, A., Ozawa, K., Yahagi, K., Hirai, H., Tsuboi, T., Kitaguchi, T., Tian, L., et al. (2020). Distinct temporal integration of noradrenaline signaling by astrocytic second messengers during vigilance. Nat. Commun. *11*, 471. https://doi.org/10.1038/s41467-020-14378-x.

Payne, S., de Val, S., and Neal, A. (2018). Endothelial-specific cre mouse models. Arterioscler. Thromb. Vasc. Biol. *38*, 2550–2561. https://doi.org/10.1161/ATVBAHA.118.309669.

Pepys, M.B., and Hirschfield, G.M. (2003). C-reactive protein: a critical update. J. Clin. Invest. *111*, 1805–1812. https://doi.org/10.1172/JCI18921.

Putnam, F.W. (1984). Progress in plasma proteins. The Plasma Proteins: Structure, Function, and Genetic Control, *Volume IV* (Academic Press Inc.), pp. 1–44.

Rungta, R.L., Zuend, M., Aydin, A.-K., Martineau, É., Boido, D., Weber, B., and Charpak, S. (2021). Diversity of neurovascular coupling dynamics along vascular arbors in layer II/III somatosensory cortex. Commun Biol. *4*, 855. https://doi.org/10.1038/s42003-021-02382-w.

Sands, M.S. (2011). AAV-mediated liver-directed gene therapy. Methods Mol. Biol. *807*, 141–157. https://doi.org/10.1007/978-1-61779-370-7_6.

Schindelin, J., Arganda-Carreras, I., Frise, E., Kaynig, V., Longair, M., Pietzsch, T., Preibisch, S., Rueden, C., Saalfeld, S., Schmid, B., et al. (2012). Fiji: an open-source platform for biological-image analysis. Nat. Methods *9*, 676–682. https://doi.org/10.1038/nmeth.2019.

Schmidt, R.F. (1989). Human Physiology (Springer-Verlag Berlin Heidelberg).

Schreiber, G., and Urban, J. (1978). The synthesis and secretion of albumin. In Reviews of Physiology, Biochemistry and Pharmacology (Springer Berlin Heidelberg), pp. 27–95.

Schulze, R.J., Schott, M.B., Casey, C.A., Tuma, P.L., and McNiven, M.A. (2019). The cell biology of the hepatocyte: a membrane trafficking machine. JCB (J. Cell Biol.) *218*, 2096–2112. https://doi.org/10.1083/jcb.201903090.

Seylaz, J., Charbonné, R., Nanri, K., von Euw, D., Borredon, J., Kacem, K., Méric, P., and Pinard, E. (1999). Dynamic *in vivo* measurement of erythrocyte velocity and flow in capillaries and of Microvessel diameter in the rat brain by confocal laser microscopy. J. Cerebr. Blood Flow Metabol. *19*, 863–870. https://doi.org/10.1097/00004647-199908000-00005.

Shaner, N.C., Lambert, G.G., Chammas, A., Ni, Y., Cranfill, P.J., Baird, M.A., Sell, B.R., Allen, J.R., Day, R.N., Israelsson, M., et al. (2013). A bright monomeric green fluorescent protein derived from Branchiostoma lanceolatum. Nat. Methods *10*, 407–409. https://doi.org/10.1038/nmeth.2413.

Shinohara, Y., Konno, A., Nitta, K., Matsuzaki, Y., Yasui, H., Suwa, J., Hiromura, K., and Hirai, H. (2019). Effects of neutralizing antibody production on AAV-PHP.B-mediated transduction of the mouse central nervous system. Mol. Neurobiol. *56*, 4203–4214. https://doi.org/10.1007/s12035-018-1366-4.

Swank, R.L., and Escobar, A. (1957). Effects of dextran injections on blood viscosity in dogs. J. Appl. Physiol. *10*, 45–50. https://doi.org/10.1152/jappl.1957.10.1.45.

Takano, T., Tian, G.-F., Peng, W., Lou, N., Libionka, W., Han, X., and Nedergaard, M. (2006). Astrocyte-mediated control of cerebral blood flow. Nat. Neurosci. *9*, 260–267. https://doi.org/10.1038/nn1623.

Takata, N., Nagai, T., Ozawa, K., Oe, Y., Mikoshiba, K., and Hirase, H. (2013). Cerebral blood flow modulation by basal forebrain or whisker stimulation can occur independently of large cytosolic Ca2+ signaling in astrocytes. PLoS One *8*, e66525.

Turner, P. v, Brabb, T., Pekow, C., and Vasbinder, M.A. (2011). Administration of substances to laboratory animals: routes of administration and factors to consider. J. Am Assoc. Lab Anim. Sci. *50*, 600–613.

Unekawa, M., Tomita, M., Tomita, Y., Toriumi, H., Miyaki, K., and Suzuki, N. (2010). RBC velocities in single capillaries of mouse and rat brains are the same, despite 10-fold difference in body size. Brain Res. *1320*, 69–73. https://doi.org/10.1016/j.brainres.2010.01.032.

Ungvari, Z., Tarantini, S., Donato, A.J., Galvan, V., and Csiszar, A. (2018). Mechanisms of vascular aging. Circ. Res. *123*, 849–867. https://doi.org/10.1161/CIRCRESAHA.118.311378.

Viecelli, H.M., Harbottle, R.P., Wong, S.P., Schlegel, A., Chuah, M.K., VandenDriessche, T., Harding, C.O., and Thöny, B. (2014). Treatment of phenylketonuria using minicircle-based naked-DNA gene transfer to murine liver. Hepatology *60*, 1035–1043. https://doi.org/10.1002/hep.27104.

Wei, H.S., Kang, H., Rasheed, I.-Y.D., Zhou, S., Lou, N., Gershteyn, A., McConnell, E.D., Wang, Y., Richardson, K.E., Palmer, A.F., et al. (2016). Erythrocytes are oxygen-sensing regulators of the cerebral microcirculation. Neuron *91*, 851–862. https://doi.org/10.1016/j.neuron.2016.07.016.

Williamson, M.R., Franzen, R.L., Fuertes, C.J.A., Dunn, A.K., Drew, M.R., and Jones, T.A. (2020). A window of vascular plasticity coupled to behavioral recovery after stroke. J. Neurosci. *40*, 7651–7667. https://doi.org/10.1523/JNEUROSCI.1464-20.2020.

Xie, J., Farage, E., Sugimoto, M., and Anand-Apte, B. (2010). A novel transgenic zebrafish model for blood-brain and blood-retinal barrier development. BMC Dev. Biol. *10*, 76. https://doi.org/10.1186/1471-213X-10-76.

Yardeni, T., Eckhaus, M., Morris, H.D., Huizing, M., and Hoogstraten-Miller, S. (2011). Retro-orbital injections in mice. Lab Anim (NY) *40*, 155–160. https://doi.org/10.1038/laban0511-155.

Zaias, J., Mineau, M., Cray, C., Yoon, D., and Altman, N.H. (2009). Reference values for serum proteins of common laboratory rodent strains. J. Am Assoc. Lab Anim. Sci. *48*, 387–390.

Zincarelli, C., Soltys, S., Rengo, G., and Rabinowitz, J.E. (2008). Analysis of AAV serotypes 1-9 mediated gene expression and tropism in mice after systemic injection. Mol. Ther. *16*, 1073–1080. https://doi.org/10.1038/mt.2008.76.

STAR★METHODS

KEY RESOURCES TABLE

REAGENT or RESOURCE	SOURCE	IDENTIFIER
Antibodies		
Rabbit anti-IBA1	FUJIFILM Wako	WAKO 019-19741, lot# WDE1198
Mouse anti-mNG	Chromotek	Cat# 32f6-100; RRID:AB_2827566
Alexa Fluor 568 goat anti-chicken	Thermo Fisher Scientific	Cat# A-11041; RRID:AB_2534098
Alexa Fluor 594 goat anti-rabbit	Thermo Fisher Scientific	Cat# A-11012; RRID:AB_2534079
Bacterial and virus strains		
pAAV-P3-Alb-mNG	This manuscript	Addgene ID: 183460
pAAV-P3-Alb-mScarlet	This manuscript	Addgene ID: 183461
pAAV-P3-Alb-Rosmarinus	This manuscript	Addgene ID: 183462
pAAV-P3-Alb-mCarmine	This manuscript	Addgene ID: 183464
pAAV-P3-IgKL-mNG	This manuscript	Addgene ID: 183465
pAAV-CBh-Alb-mNG	This manuscript	Addgene ID: 183466
pAAV-CBh-IgKL-mNG	This manuscript	Addgene ID: 183467
pZac2.1 GfaABC1D NAPA-A SV40	Addgene	Addgene ID: 92281
pCS2+mNeonGreen-C Cloning Vector	Addgene	Addgene ID: 128144
pmScarlet_C1	Addgene	Addgene ID: 85042
pAAV/CBh_*-WPRE-SV40pA	Viral Vector Core, Gunma University, Initiative for Advanced Research	N/A
pAAV-P3-EGFP	Viral Vector Facility, University of Zurich	p438; https://www.vvf.uzh.ch/en.html
Chemicals, peptides, and recombinant proteins		
Lipopolysaccharides from *E. coli*	Sigma-Aldrich	L8274
VECTASHIELD DAPI	Vector laboratories	H-1200-10
Texas Red dextran 70k MW	Invitrogen	D1830
Critical commercial assays		
Mouse Albumin ELISA kit	Abcam	ab108792
Mouse CRP ELISA Kit	Thermo Fisher	EM20RB
Deposited data		
Raw and Analyzed data	This manuscript	https://doi.org/10.6084/m9.figshare.20629626
Experimental models: Cell lines		
HEK293T	Dharmacon	HCL4517
Experimental models: Organisms/strains		
Mus musculus: C57BL/6JRj	Javier	N/A
Software and algorithms		
Fiji (ImageJ)	(Schindelin et al., 2012)	http://fiji.sc
Matlab 2019b	Mathworks, USA	https://www.mathworks.com/products/matlab.html
Graphpad Prism 9	GraphPad	https://www.graphpad.com/scientific-software/prism/

RESOURCE AVAILABILITY

Lead contact

Further information and requests for resources and reagents should be directed to and will be fulfilled by the lead contact, Hajime Hirase (hirase@sund.ku.dk).

Materials availability

All plasmids generated as part of this study have been deposited at Addgene: pAAV-P3-Alb-mNG (#183460), pAAV-P3-Alb-mScarlet (#183461), pAAV-P3-Alb-Rosmarinus (#183462), pAAV-P3-Alb-mCarmine (#183464), pAAV-P3-IgKL-mNG (#183465), pAAV-CBh-Alb-mNG (#183466), pAAV-CBh-IgKL-mNG (#183467).

Data and code availability

- All data used to generate the figure of this study have been deposited at figshare and are publicly available as of the date of publication. DOIs are listed in the key resources table.
- This paper does not report original code.
- Any additional information required to reanalyze the data reported in this work paper is available from the lead contact upon request.

EXPERIMENTAL MODEL AND SUBJECT DETAILS

C57BL/6JRj mice (Javier) of either sex in an age range of 1.5–6 months were used. Mice were housed in 12-h light/12-h dark cycle (lights on: 7 a.m.) with food and water *ad libitum*. The procedures involving animal care, surgery, *in vivo* imaging, and sample preparation were approved by the local research ethics committee (Department of Experimental Medicine, University of Copenhagen) and conducted in accordance with the Danish Animal Experiments Inspectorate.

METHOD DETAILS

DNA constructs

Mouse albumin (*Alb*) nucleotide sequence was obtained from the NIH nucleotide database (NCBI Reference Sequence: NM_009654.4). The IgK leader (*IgKL*) and mNeonGreen (mNG) nucleotide sequences were obtained from the Addgene web site (plasmids 92,281 and 128144, respectively). mNG-Alb was constructed by concatenating the Alb and mNG sequences with the linker sequence SmaI-scFv-AgeI, where scFV represents the (Gly4Ser) x3 amino acid sequence coded by GGT GGA GGC GGT TCA GGC GGA GGT GGC TCT GGC GGT GGC GGA TCA. Likewise, Alb-mNG was constructed by concatenating the mNG and Alb sequences with the linker sequence SmaI-scFv-SalI. The construction for secretory mNG protein IgKL-mNG achieved by concatenation of a shortened IgKL signal peptide (MTDTLLLWVLLLWVPGSTGD) to mNG. mNG-Alb, Alb-mNG, and IgKL-mNG were artificially synthesized and cloned into a mammalian expression vector (Twist Bioscience, pTwist CMV Betaglobin WPRE Neo). For all fusion protein constructs, the first methionine codon was removed from the second protein sequence. The tertiary structure of Alb-mNG was predicted by the Phyre2 program using the intensive mode (Kelley et al., 2015).

pAAV-CBh-Alb-mNG and pAAV-CBh-IgKL-mNG were constructed by ligating the insert to the AAV backbone vector pAAV/CBh_*-WPRE-SV40pA (Viral Vector Core, Gunma University Initiative for Advanced Research) via the AgeI and NotI sites. pAAV-P3-Alb-mNG and pAAV-P3-IgKL-mNG were made using pAAV-P3-EGFP as a template (p438, Viral Vector Facility VVF, Institute of Pharmacology and Toxicology, University of Zurich). pAAV-P3-Alb-mScarlet was made by replacing mNeonGreen with mScarlet (sequence from Addgene plasmid #85042 with a silent mutation to eliminate the NotI site within the mScarlet cDNA). The artificially synthesized DNA segment containing the partial sequence Alb-scFV and mScarlet was subcloned into pAAV-P3-Alb-mNeonGreen via NdeI and EcoRI. AAVs encoding Alb-mNG or IgKL-mNG were produced using the ultracentrifugation method as described previously (Konno and Hirai, 2020). The titers of purified AAVs were as follows: AAV8-P3-Alb-mNG (3.99×10^{13} vg/mL), AAV8-P3-IgKL-mNG (2.67×10^{13} vg/mL), AAV8-P3-Alb-mScarlet (1.65×10^{13} vg/mL). AAV8-P3-EGFP was obtained from VVF (v438, 4.5×10^{12} vg/mL).

Cell culture

HEK293T cells (Dharmacon, HCL4517), cultured in DMEM supplemented with 10% FBS and 50 U/mL penicillin-streptomycin (Thermo Fisher Scientific, 41965039, 16141079 and 15140122), were transfected in a 24-well plate using Fugene HD (Promega, E2311) at 30% confluency. For each well, transfection reagent was mixed with 0.5 μg of plasmid DNA at a 3:1 ratio (μL/μg) in 25 μL Opti-MEM (Thermo Fisher Scientific, 31985070) and added dropwise after 15-minute incubation at room temperature. Each transfection was carried out in six replicates. Cells were imaged under the microscope (Nikon Eclipse Ti) at 24 h and 48 h after transfection. To evaluate secretion of expressed molecular tracers, the ratio of extracellular and cytosolic fluorescence intensity was calculated. To further quantitate the secretion of molecular tracers, 200 μL of culture medium was collected from each well and centrifuged for five minutes at 1200 rpm, 100 μL of the supernatant was subjected to fluorescent measurements in a black 96-well plate (Thermo Fisher Scientific, 437796) using a SpectraMax iD3 microplate reader (Molecular Devices, excitation/emission at 485/538 nm). The cell culture medium was collected 24 h and 48 h after transfection from separate sets of cells.

In vivo recombinant protein expression

Long-term *in vivo* transgenes expression in the liver was achieved by systemic administration of AAV, up to 6×10^{11} vg in 0.3–0.6 mL sterile phosphate buffered saline (PBS). Intraperitoneal injection (i.p.), or intraveneous injection (i.v.) via the tail vein or retro-orbital sinus was performed. For tail vein i.v. injection, mice were briefly anesthetized with isoflurane (~1-2%) and mounted in a stereotactic frame. Retro-orbital injections were performed according to the protocol by Yardeni and colleagues (Yardeni et al., 2011). after a brief anesthesia with isoflurane. Mice were recovered in the home cage thereafter. Other *in vivo* transfection methods such as hydrodynamic transfection using pCAG DNA plasmids nor liposome-based transfection using a commercial reagent did not result in sufficient or sustained expression for the detection of fluorescence in the plasma.

Biochemical analysis

Blood sampling and plasma extraction

To prevent clotting of the blood, heparin (500U, LEO) was injected i.p. to deeply anesthetized mice thirty minutes prior to perfusion-fixation. Total of 0.5–0.7 mL blood was collected from the heart and stored in 0.75 mL tubes containing 5 μL EDTA and 5 μL Halt protease and phosphatase inhibitor cocktail (100x, Thermo Scientific). The tubes were centrifuged for 10 min (2000 x G, 4°C), and the supernatant was collected as plasma. Plasma samples were stored in aliquots at −80°C until further imaging and analysis.

Plasma albumin quantification

Total plasma albumin concentration was determined using a commercially available enzyme-linked immune sorbent assay (ELISA) kit (Abcam, ab108792). The ELISA was performed according to the manufacture's protocol and the results were measured using a SpectraMax iD3 microplate reader (OD 450 nm). Sample duplicates were measured in 1:2,000,000 dilution and concentrations were calculated with Microsoft Excel using a four-parameter logistic curve-fit as recommended by the manufacturer.

Plasma mNG quantification

The mNG protein was purified from an *E.coli* expression system (Gene Universal, USA) and was reconstituted at varying concentrations ranging from 6.25 to 200 nM in PBS for calibration of mNG concentration (standard curve, triplicates). Plasma samples of Alb-mNG expressing mice were diluted at 1:10 in PBS. The fluorescence of samples was measured in duplicates using a SpectraMax iD3 plate reader (excitation 485 nm, emission 535 nm). mNG concentrations in plasma samples were calculated by the standard curve (linear fit).

Estimation of relative Alb-mNG proportion

The relative proportion of Alb-mNG in plasma samples was calculated from the assay results of total albumin and mNG concentrations. The albumin concentration was converted to molar concentration using the albumin molecular weight of 65.9 kDa. Then we divided the molar values of mNG by the molar values of albumin to obtain the relative proportion of total mNG for each sample. The relative proportion was plotted as percentage of total albumin concentration.

Plasma C-reactive protein assessment

To examine possible systemic inflammation in response to viral expression of Alb-mNG, plasma C-reactive protein (CRP) was measured using an ELISA (Invitrogen, EM20RB). The ELISA was performed according to the manufactures protocol and the samples were measured using a SpectraMax iD3 microplate reader (OD 450 nm). In brief, sample duplicates were measured in 1:2000 dilutions. CRP concentrations were calculated using a four-parameter logistic curve-fit as recommended.

Ex vivo *macro fluorescence imaging*

To examine the development of fluorescent tracer expression in the same animals over a time course of 8 weeks, blood was sampled from the tail in borosilicate glass capillaries (1B100F-4 or 1B150F-4, WPI) and examined by a macroscope (Leica M205 FA) equipped with an X-Cite 200Dc light source, digital camera (C11440 Orca-flash 4.0, Hamamatsu). Filter sets ET GFP LP (excitation 480/40, emission 510LP, 10447407, Leica), ET mCherry (excitation 560/40, emission 630/75m, 10450195, Leica), and ET CY5 (excitation 620/60, emission 700/75, 10447413, Leica) were used to image green, red/deep red, and cyan, respectively. Images were acquired using Leica Application Suite X software (version 2.0.0.14332.2).

Open field test

The open field arena was a square of 40 x 40 cm^2 white foam polyvinyl chloride (PVC) box. The inner area of 24 x 24 cm^2 is considered as arena center. Mice were handled for 5 min each day, for at least 3 days before the experiment. On the day of the experiment, mice were transferred to the testing room at least 2 hours before testing and tested at the end of the light cycle (5–7 pm). Mouse movement was recorded with a video camera placed above the open field box. The test was initiated by placing a single mouse at the corner of the box, thereafter the mouse explored the arena freely for 10 min. Only the last 6 min of the 10 min recording were used for the data analysis. The box was cleaned with alcohol and water after each session.

Histology

Deeply anesthetized mice (ketamine/xylazine 100 mg/kg and 20 mg/kg, respectively) were transcardially perfused with physiological saline briefly followed by 4% paraformaldehyde (PFA) in 0.1 M phosphate buffer (PB, pH 7.4) using a peristaltic pump. To induce an inflammatory state for histological positive controls a subset of mice received a single dose of lipopolysaccharides (10 mg/kg, Sigma-Aldrich, L8274) 24h prior to perfusion. Body organs including liver and brain were harvested and post-fixed in 4% PFA overnight before further storage in PBS. 50 μm sections were prepared using a vibratome (Leica VT1200 S) in PBS. Brain and liver sections

were incubated with a rabbit anti-IBA1 (WAKO 019-19741, lot# WDE1198, 1:1000) to assess microglial for hepatic macrophage morphology. Anti-mNG (chromotek 32F6, 1:1000) was used for the detection of mNeonGreen expression.

Primary antibodies were detected using the following secondary antibodies: Alexa Fluor 568 goat anti-chicken (Thermo Fisher Scientific, A11041, 1:1000), Alexa Fluor 594 goat anti-rabbit (Thermo Fisher Scientific, A11012, 1:1000), Alexa Fluor 568 goat anti-rat (Abcam AB1755710, 1:1000). Stained tissue slices were mounted with antifade mounting medium with DAPI (Vector laboratories, Vectashield, H-1200). Images were acquired using a standard fluorescence microscope (Nikon ECLIPSE Ni-E) and a digital camera (Mono-Camera Nikon DS-Fi3) controlled by an imaging software (NIS-Elements Imaging software AR 4.60.00). Confocal images were acquired by a Nikon Eclipse Ti2 microscope with a Plan Apo x60/1.40 numerical aperture (NA) oil objective controlled by an imaging software NIS-Elements AR 4.50.00.

In vivo fluorescence imaging

Cranial window surgery

Mice were anesthetized by 3-4% isoflurane for induction and then mounted to the stereotaxic frame. Throughout the surgery, the anesthesia was maintained at 1–1.5% isoflurane and the body temperature was maintained at 37 °C with a heating pad. The skull was exposed after applying local analgesia (lidocaine, 0.2 mg/mL) by making an incision to the scalp, and a metal frame (head plate) was then attached to the skull using dental cement (Super Bond C&B, Sun Medical, Shiga, Japan). A 4-mm diameter craniotomy above the somatosensory cortex was made and the dura mater was surgically removed. 4mm diameter autoclaved cover slip was carefully mounted to cover the brain and then sealed by dental cement. Mice received a subcutaneous administration of carprofen (5 mg/kg) for systemic analgesia after the surgery and were recovered in their home cage.

Two-photon microscopy

Two-photon imaging were performed on anesthetized (70 mg/kg ketamine, 10 mg/kg xylazine) or awake mice. For awake mouse imaging, mice were acclimatized to head fixation at least a week before the imaging experiments (MAG-1 or MAG-2, Narishige). To mount a mouse for awake imaging, the mouse was briefly anesthetized by 2% isoflurane and head plate fixation was secured in the microscopy apparatus. Imaging session started twenty minutes after the mouse was mounted under the objective lens.

The two-photon microscope setup consisted of a B-Scope (Thorlabs) equipped with a resonant scanner, a Chameleon Vision 2 laser (Coherent), an objective lens (Apo LWD 25×/1.10w; CFI75 Apo 25XC W, Nikon), and the primary dichroic mirror ZT405/488/561/680-1100rpc (Chroma) as described before (Oe et al., 2020). Emission light was separated by the secondary dichroic mirror (FF562-Di03, Semrock) with band-pass filters FF03-525/50 and FF01-607/70 (both Semrock). mNG and mScarlet were excited at 940 nm and 1000 nm, respectively. For simultaneous imaging of mNG and Texas Red, FF01-647/70 (Semrock) was used for the red channel to avoid bleedthrough and the excitation wavelength was set at 950 nm. Images were acquired using ThorImage Software Version 3.0. The laser power under the objective lens was measured by a power meter (Thorlabs) before imaging to ensure consistent excitation across chronic monitoring of plasma tracer.

Comparison of plasma Alb-mNG and Texas Red dextran was made using mice under anesthesia. After baseline volumetric imaging, Texas Red dextran (70k MW, D1830, Invitrogen) was administered i.v. (retro-orbital, 15 mg/mL in saline, 50 μL). Linear scaling of laser power with imaging depth was applied (power under the objective lens: 10–35 mW for 0–500 μm). Successive imaging was performed at 10, 60, and 120 min.

Functional hyperemia imaging was performed with awake mice. Imaging and sensory stimulation were synchronized via a pulse generator (Master-9, A.M.P.I) connected to the B-Scope hardware. Whisker-evoked functional hyperemia in the barrel cortex was induced by presenting air puffs (50 ms duration, 10 Hz, 5 s, 30 PSI) to the mouse's whisker pad 30 and 65 s after the start of imaging. Each imaging session lasted at least for 70 s.

Capillary flow was captured by restricting the imaging area to a single capillary with non-averaged bidirectional scanning, achieving frame rates of up to 220 Hz. The excitation power under the objective lens was kept under 20 mW.

Fluorescence imaging by macroscope

The same macroscope for glass capillary imaging (Leica M205 FA) was used for imaging of coverslipped cranial window of AAV-injected mice. Briefly, aneasthetised mice were head fixed to a MAG-1 or MAG-2 headplate fixture apparatus and placed under the macroscope. Filter sets ET GFP LP (excitation 480/40, emission 510LP, 10447407, Leica), ET mCherry (excitation 560/40, emission 630/75m, 10450195, Leica), and ET CY5 (excitation 620/60, emission 700/75, 10447413, Leica) were used to image green, red/deep red, and cyan, respectively.

QUANTIFICATION AND STATISTICAL ANALYSIS

Image data analysis

To calculate the relative intensity between intracellular and extracellular fluorescence (Figure 1D), cellular areas were detected by an adaptive threshold approach using the *imbinarize()* function in Matlab (Mathworks, USA) on greyscale-converted cell culture images. The mean intensity was calculated for the cellular area and compared with the extracellular mean intensity. Identical excitation intensity and exposure time was used for all time-points analyzed.

Vascular fluorescence for Texas Red dextran and Alb-mNG (Figures 3B and 3C) was calculated by first detecting vascular areas using the Otsu thresholding method applied to the 3D image stack using the *graythresh()* Matlab function. Mean intensity was

calculated for the detected vascular areas. To compare different time points, mean intensity signals were normalized to the time point at 10 min after Texas Red dextran injection for each channel.

Red blood cell velocity (Figure 3F) was estimated by computing the unbiased cross-correlogram of the intensity signals from two distant locations on the same capillary (Chaigneau et al., 2003). The time-intensity vectors of the two chosen points were transformed to z values to compute the correlation coefficients.

Arterial diameter dynamics for functional hyperemia experiments (Figures 3H–3J) were determined as follows. First, the intensity profile along a manually selected line that intersects the target vessels was computed using the *improfile()* function in Matlab. The edges of the vessels were estimated by detecting the sharp intensity signal decreases, and the vessel diameter is calculated as the distance between the two edges. Computed vascular diameter function was normalized to the mean diameter during a 2-s period before sensory stimulation.

Signal-to-noise ratio (Figure 4B) was calculated as the ratio of vasculature fluorescence minus the parenchymal fluorescence divided by the parenchymal fluorescence. Vasculature was identified as previously, and mean signal was calculated as well as for the extra-vascular parenchyma. For Shannon's entropy calculation, the image matrices were first converted to probability matrices by dividing by their total single intensities and custom function *info_entropy()* was used (Vallabha Hampiholi; Entropy Calculator; MATLAB Central File Exchange).

Statistical analysis

All measured values are indicated as mean ± SEM. Comparisons of two sample group means were assessed by t-test. Multiple group comparisons were performed using one-way or two-way ANOVA unless otherwise noted. Graph Prism 9 was used for all statistical analyses.

Cell Reports Methods, Volume *2*

Supplemental information

Liver-secreted fluorescent blood plasma markers enable chronic imaging of the microcirculation

Xiaowen Wang, Christine Delle, Antonis Asiminas, Sonam Akther, Marta Vittani, Peter Brøgger, Peter Kusk, Camilla Trang Vo, Tessa Radovanovic, Ayumu Konno, Hirokazu Hirai, Masahiro Fukuda, Pia Weikop, Steven A. Goldman, Maiken Nedergaard, and Hajime Hirase

Supplemental information

Liver-secreted fluorescent blood plasma markers enable chronic imaging of the microcirculation

Xiaowen Wang[1+], Christine Delle[1+], Antonis Asiminas[1+], Sonam Akther[1], Marta Vittani[1], Peter Brøgger[1], Peter Kusk[1], Camilla Trang Vo[1], Tessa Radovanovic[1], Ayumu Konno[2,3], Hirokazu Hirai[2,3], Masahiro Fukuda[4,5], Pia Weikop[1], Steven A Goldman[1,6], Maiken Nedergaard[1,6*], Hajime Hirase[1,6,7*]

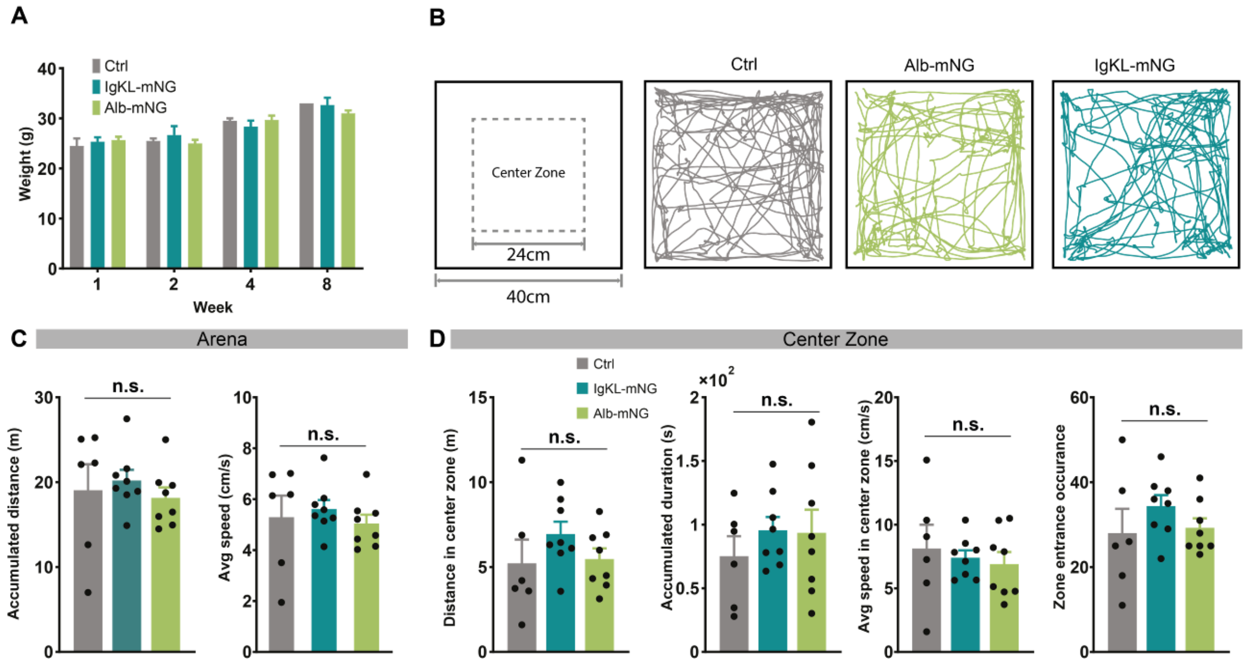

Figure S1. Plasma tracer expression does not display obvious phenotypes in body weight or open field behavior. Related to Figure 2. (A) Body weight of control (age matched sham) and AAV-P3-IgKL-mNG-injected and AAV8-P3-Alb-mNG-injected mice during 1 to 8 weeks post-injection. AAV-injected mice show no differences in body weight compared to control; two-way ANOVA: significant main effect of time, no significant effect of group, or group x time interaction; n=3 mice per group. (B) Schematic of the arena used for open field test and example traces of mouse trajectory for the last 6 min of 10 min recording. (C) Total distance traveled (left) and mean speed of movement (right) during the last 6 min of open field behavior; one-way ANOVA: no significant main effect of group; n=6–8 mice per group. (D) Metrics on center zone behavior. Distance moved, total time, speed of movement, and frequency of visiting the center zone did not show significant differences among control and AAV injected mice; one-way ANOVA: no significant main effect of group for all metrics $p>0.05$; n=6–8 mice per group. All graphs show means ± SEM.

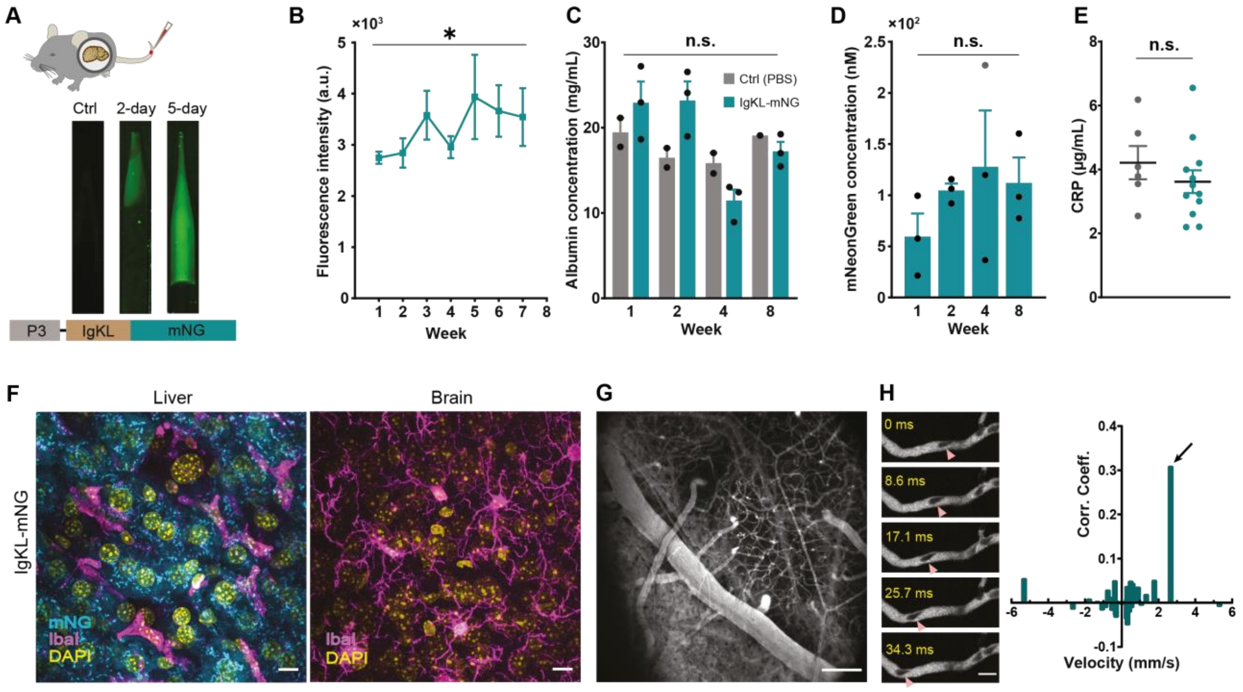

Figure S2. Liver-targeted expression IgKL-mNG (secretory mNG). Related to Figure 2. (A) A secretory form of mNeonGreen, IgKL-mNG, is expressed in the liver by systemic injection of AAV8-P3-IgKL-mNG in mice. Fluorescence signals were detected in the blood samples two days after AAV injection. (B) Chronic monitoring of plasma fluorescence. Note that the plasma intensity is an order of magnitude lower than Alb-mNG (Fig. 2) (one-way ANOVA: significant effect of time, $p < 0.05$); n=6 mice. (C) Plasma albumin concentration and plasma mNG concentration (D) over eight weeks; Albumin concentration: two-way ANOVA: no significant effect of time, group or interaction; mNG concentration: one-way ANOVA: no significant effect of time; n=3 mice. (E) CRP levels during the 8 weeks of post-AAV injection period is indistinguishable from sham-injected control. (t-test, $p > 0.05$; $n_{control}=6$, $n_{IgKL\text{-}mNG}=12$. (F) Liver and brain images 3 weeks after AAV injection. Immunofluorescence: mNG (cyan), IBA1 (mangenta), DAPI (yellow). Scale bar 10 µm. (G) Two-photon imaging through a cranial window visualizes cerebral blood vasculature despite the relatively low fluorescence signal intensity. (H) Capillary blood flow is also quantifiable using IgKL-mNG as a plasma tracer (RBC speed = 2.65 mm/s). All graphs show means ± SEM; * $p < 0.05$.

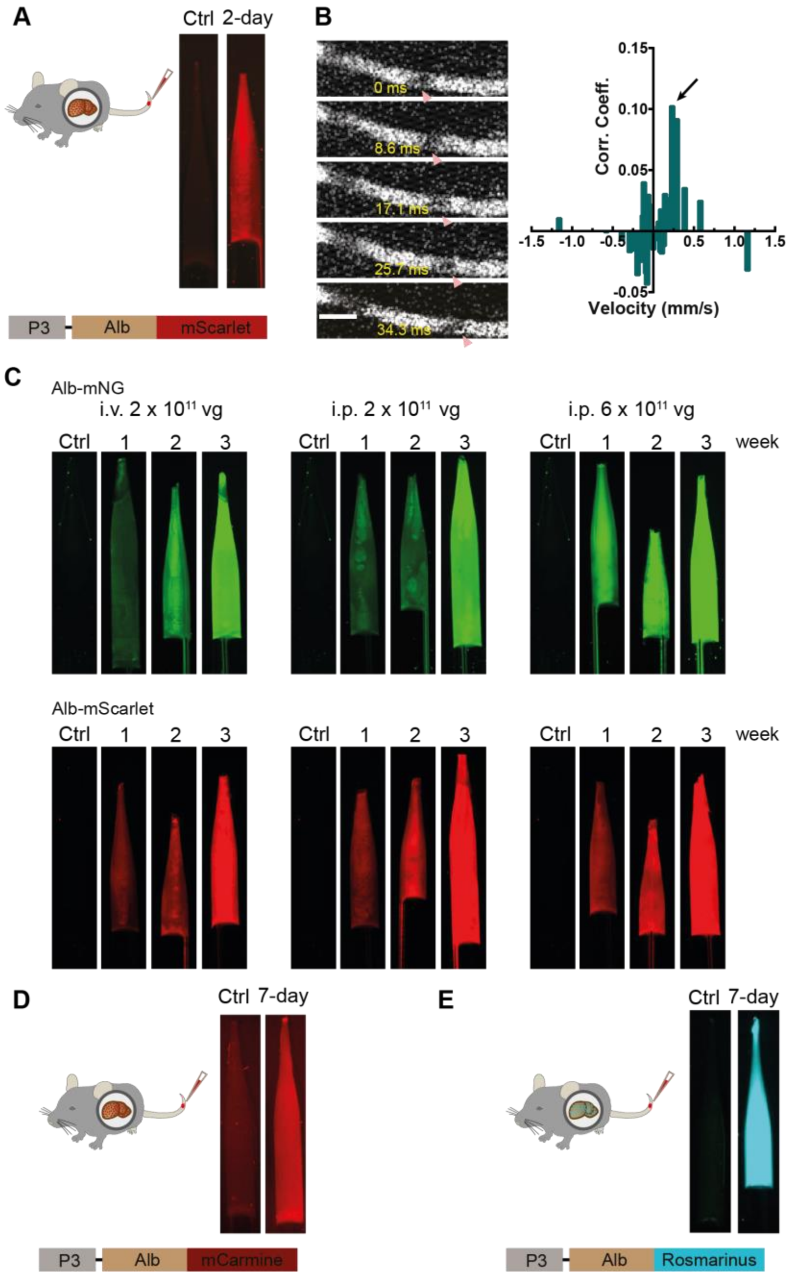

Figure S3. Expanding the spectrum of liver-secreted plasma fluorescent probes. Related to Figures 2, 3 and 4. (A) Retro-orbital i.v. injection of AAV8-P3-Alb-mScarlet results in labeling of blood plasma with red fluorescence, thereby representing a plasma tracer that is spectrally distinct from Alb-mNG. (B) Capillary flow dynamics is reliably visualized by two-photon microscopy (RBC speed = 0.25 mm/s). (C) Samples of fluorescent blood plasma taken from animals that were injected either i.v. or i.p. (at two different concentrations) with either AAV8-P3-Alb-mNG or AAV8-P3-Alb-mScarlet 1, 2, and 3 weeks after injection. (D–E) Alb-based plasma tracer spectrum is further extended by the addition of and Alb-mCarmine (D: deep red fluorescence), and Alb-Rosmarinus (E: cyan fluorescence). For both, example of the fluorescence signals in blood samples collected on day 7 after a single retro-orbital i.v. injection of either AAV8-P3-Alb-mCarmine or AAV8-P3-Alb-Rosmarinus.

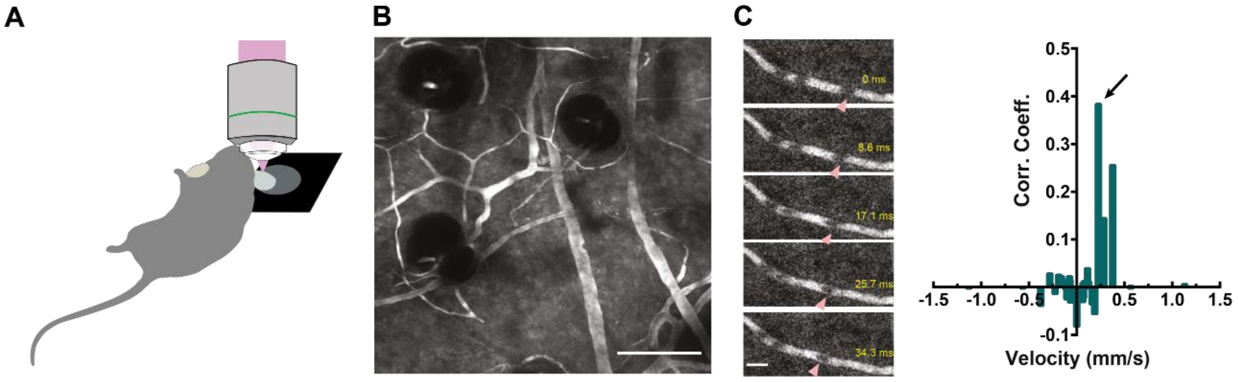

Figure S4. Alb-mNG is suitable for studying vasculature in peripheral tissues. Related to Figure 3. (A) Schematic of two-photon imaging of the ear skin capillary network in an Alb-mNG expressing mouse under ketamine-xylazine anesthesia. (B) Example image of ear vasculature. The black holes are the cavity space for hair follicle. Scale bar 100 µm (C) Measurement of blood flow in peripheral ear capillary via two-photon imaging (RBC speed = 0.23 mm/s). Scale bar 10 µm.

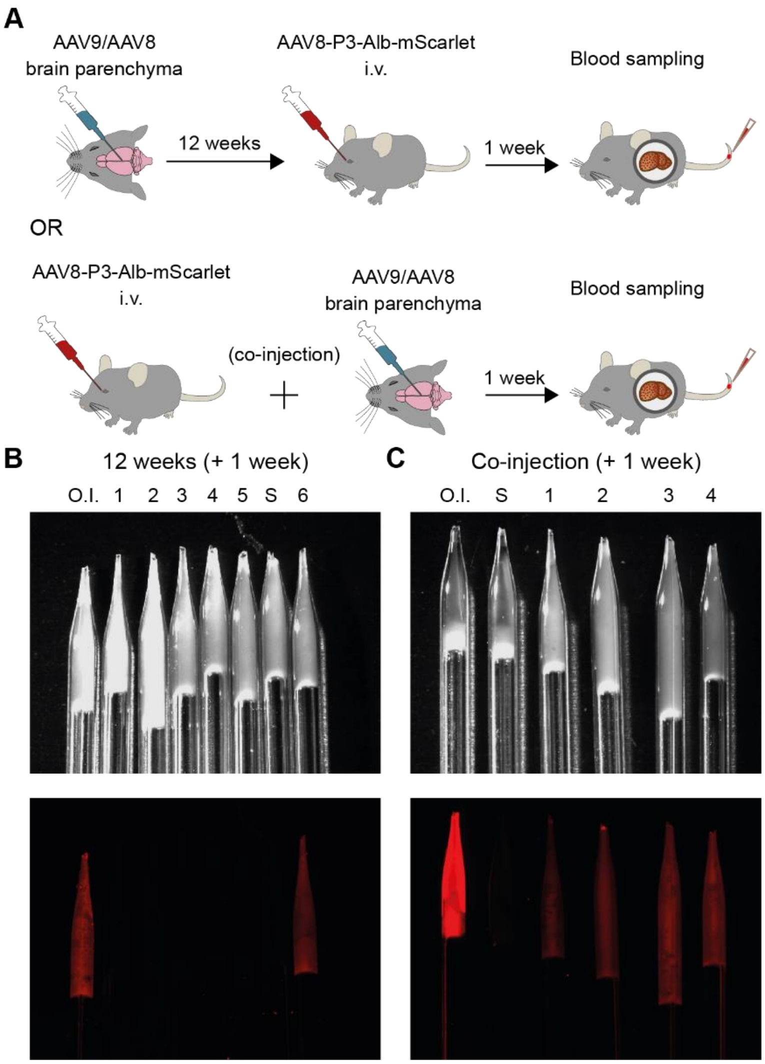

Figure S5. Expression of albumin-fused fluorescent probes is contigent on time after first AAV injection directly into brain parenchyma. Related to Figure 2 and 3. (A) Schematic of procedures. Mice were injected directly into parenchyma with AAV9-hSyn-GRAB$_{NE2m}$ and AAV8-hSyn-GRAB$_{ACh3.0}$ followed by retro-orbital administration (i.v.) of AAV8-P3-Alb-mScarlet 12 weeks after (top) or injected retro-orbitally (i.v.) with AAV8-P3-Alb-mScarlet and within 1 h were injected directly into parenchyma with AAV9-hSyn-GRAB$_{NE2m}$ and AAV8-hSyn-GRAB$_{ACh3.0}$ (bottom). (B) Bright-field (top) and red fluorescence images (bottom) of glass capillaries containing blood samples collected 1 week after i.v. injection of AAV8-P3-Alb-mScarlet proceeded by 12 weeks of brain parenchymal injection of AAV9-hSyn-GRAB$_{NE2m}$ and AAV8-hSyn-GRAB$_{ACh3.0}$ in five mice (1–5). A mouse injected with AAV8-P3-Alb-mScarlet 4 weeks before blood sampling (O.I.) and a mouse injected only with AAV8-P3-Alb-mScarlet (6) were used as positive controls. Blood from a saline-injected mouse was used as a negative control (S). (C) Bright-field (top) and red fluorescence images (bottom) of glass capillaries containing blood samples collected 1 week after co-injection of AAV8-P3-Alb-mScarlet (i.v.) and brain parenchymal AAV9-hSyn-GRAB$_{NE2m}$ and AAV8-hSyn-GRAB$_{ACh3.0}$ in four mice (1–4). A mouse injected with AAV8-P3-Alb-mScarlet 4 weeks before blood sampling (O.I.) was used as a positive control and blood from a saline-injected mouse was used as a negative control (S).
